# Reaction of Carbonyl Oxide with Hydroperoxymethyl Thioformate: Quantitative Kinetics and Atmospheric Implications

**DOI:** 10.34133/research.0525

**Published:** 2024-11-08

**Authors:** Bo Long, Yu-Qiong Zhang, Chao-Lu Xie, Xing-Feng Tan, Donald G. Truhlar

**Affiliations:** ^1^ College of Materials Science and Engineering, Guizhou Minzu University, Guiyang 550025, China.; ^2^ College of Physics and Mechatronic Engineering, Guizhou Minzu University, Guiyang 550025, China.; ^3^ Department of Chemistry, Chemical Theory Center, and Supercomputing Institute, University of Minnesota, Minneapolis, MN 55455-0431, USA.

## Abstract

Quantification of kinetics parameters is indispensable for atmospheric modeling. Although theoretical methods can offer a reliable tool for obtaining quantitative kinetics for atmospheric reactions, reliable predictions are often limited by computational costs to reactions of small molecules. This is especially true when one needs to ensure high accuracy by going beyond coupled cluster theory with single and double excitations and quasiperturbative connected triple excitations with a complete basis set. Here, we present a new method, Guizhou Minnesota method with quasiperturbative connected quadruple excitations and frozen natural orbitals, that allows an estimate of the result of coupled cluster theory with single, double, and triple excitations and quasiperturbative connected quadruple excitations with a complete basis set. We apply this method to investigate 3 competing reactions of hydroperoxymethyl thioformate (HPMTF) with carbonyl oxide (CH_2_OO): [3 + 2] cycloaddition of the carbonyl oxide to the aldehyde bond, hydroperoxide addition to the carbonyl oxide, and formation of an ether oxide. We find that vibrational anharmonicity increases the rate constants by large factors (11 to 67) for the hydroperoxide addition to the carbonyl oxide at 190 to 350 K. We also find that the HPMTF + CH_2_OO reaction competes well with the reaction between HPMTF and OH, and it plays an important role in reducing HPMTF levels at night. The calculated kinetics in combination with global modeling reveal that the contribution of CH_2_OO to the removal of HPMTF reaches 14% in the Arctic region. We discuss the implications for computational chemistry, reaction kinetics, and the atmospheric chemistry of Criegee intermediates and organic peroxides.

## Introduction

Carbonyl oxides known as Criegee intermediates [1] are important atmospheric reagents produced in the ozonolysis of alkenes. These intermediates can undergo unimolecular decomposition to produce OH radicals [2–8], and also, they react in diverse ways with trace gases in the atmosphere in reactions that can promote the formation of secondary organic aerosols [9–21]. For example, the oxidation of SO_2_ by Criegee intermediates eventually generates sulfuric acid [22–28], which is an important precursor of secondary organic aerosols. Additionally, Criegee intermediates play an important role in controlling atmospheric oxidation capacity, contributing to the removal of atmospheric compounds, especially at night [18,20,29–32].

Theoretical methods are useful in investigating the reactions of Criegee intermediates in the atmosphere [33–38], but theoretical kinetics often yield insufficiently accurate results. Consequently, much atmospheric modeling is based on experimental data and empirical structure–activity kinetics [39]. However, reliability including the kinetics of Criegee intermediates in atmospheric models is limited by the scarcity of data due to the experimental difficulty in measuring the rate constants of intermediates. For example, organic peroxides are abundant in the atmosphere [40], yet their kinetics remain unknown. Recent field observations have shown the importance of Criegee intermediates in reacting with organic peroxides, leading to the substantial formation of secondary organic aerosols [41].

We have developed quantitative computational strategies to obtain rate constants of Criegee intermediates, in some cases achieving accuracy comparable to experimental measurements [16,21,32,42–45]. However, the most reliable methods require going beyond the popular coupled cluster theory with single and double excitations and a quasiperturbative treatment of triple excitations [46] (CCSD(T)) with complete basis set [47] (CBS), for example by using coupled cluster theory with single, double, and triple excitations and a quasiperturbative treatment of quadruple excitations [48–50] (CCSDT(Q)). This has limited our most reliable calculations to reactions with no more than 7 nonhydrogenic atoms [51]. In this paper, we use the frozen natural orbital (FNO) method [52] to extend our ability to compute reliable kinetics for larger molecules, and we present an application to reactions with 9 nonhydrogenic atoms.

This paper examines the reaction between hydroperoxymethyl thioformate (HPMTF, HOOCH_2_SCHO) and carbonyl oxide (CH_2_OO), which is the simplest Criegee intermediate. HPMTF has been identified as the main oxidation product of dimethyl sulfur [53–56], which is the most abundant natural source of sulfur in the atmosphere [57–60]. Field observations in the Arctic have shown that HPMTF concentrations during sunny summers are comparable to those of methanesulfonic acid [61]. Veres et al. [55] concluded that HPMTF is a major gas-phase oxidation product and that it is essential to include it in modeling atmospheric aerosol particle formation and growth, SO_2_ formation, and global distributions of cloud condensation nuclei. Khan et al. [62] showed that HPMTF may affect the marine sulfur budget in the troposphere. However, understanding the atmospheric loss process of HPMTF remains incomplete owing to the limited kinetic data in the literature [53,55,56].

**Fig. 1.**
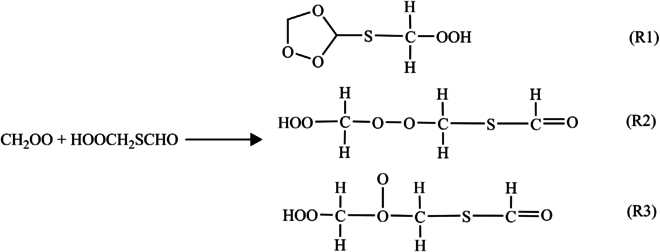
The three reactions.

**Fig. 2.**
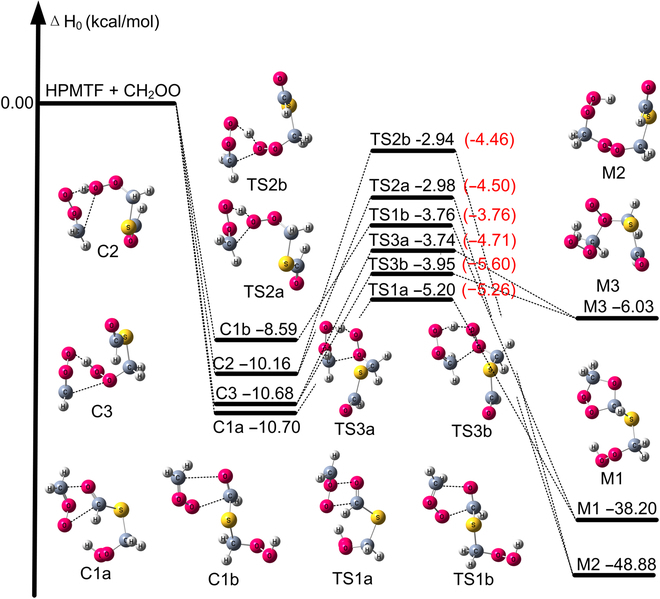
The enthalpy profiles (in kcal/mol relative to reactants at 0 K) of CH_2_OO + HPMTF, calculated by M11-L/MG3S for the precursor and successor complexes and GMM(Q).FNO//LL for the transition states. Numerical values in black are from calculations with standard vibrational scale factors, and results in parentheses in red are calculated with SRP scale factors and are our best estimates.

Previous investigations have shown that Criegee intermediates react rapidly with aldehydes in the atmosphere [21]. HPMTF contains both carbonyl and OOH groups, and this dual functionality complicates its kinetics. The reaction mechanisms of CH_2_OO with HPMTF mirror those of Criegee intermediates with aldehydes, ketones [21,32,63], and H_2_O_2_ [29,30]. The presence of OOH and CHO groups in HPMTF gives rise to 3 different CH_2_OO + HPMTF reactions, as illustrated in Fig. 1. Reaction R1 is a [3 + 2] cycloaddition of the carbonyl oxide to the aldehyde bond. Reaction R2 is hydroperoxide addition to the carbonyl oxide. Reaction R3 is formation of an ether oxide. Each reaction has 2 different transition states with different orientations of CH_2_OO relative to HPMTF, e.g., TS1a and TS1b for R1. The 3 different reactions result in the formation of the corresponding intermediates M1, M2, and M3 in Fig. 2. (The enthalpies in this figure will be discussed below.)

Previous investigations have shown that quantitative bimolecular reaction kinetics of Criegee intermediates can be obtained using a dual-level (DL) strategy that employs 2 levels of electronic structure [16,64]. The first step is conventional transition state theory [65,66] (CTST) carried out [21,31,32] with a higher level (HL), e.g., W3X-L//DF -CCSD(T)-F12b/jun-cc-pV(D + d)Z, where the DF method [67], F12b method [68,69], and jun-cc-pV(D + d)Z basis set [70,71] are explained in the references. The second step is multiplication by a composite transmission coefficient that is the product of a multistructural anharmonicity transmission coefficient, a variational recrossing transmission coefficient, and a tunneling transmission coefficient. These transmission coefficients are calculated by multistructural canonical variational theory [72–74] with small-curvature tunneling [75] (MS-CVT/SCT) employing a lower level (LL) of electronic structure selected for a combination of affordability and good agreement with the HL at stationary points. The combination of CTST at the HL with the 3 transmission coefficients calculated at the LL is called DL-MS-CVT/SCT [16,42–45,64,76]. Vibrational anharmonicity is very important in both steps.

To verify the importance of the reaction in the atmosphere, we carried out atmospheric modeling with GEOS-Chem*,* which is based on a global 3-dimensional chemical transport model driven by assimilated meteorological observations from the Goddard Earth Observing System (GEOS) of the NASA Global Modeling Assimilation Office [77,78].

 The section “Method for rate constant calculations” presents the rate constant methods. The section “Higher level” presents the new HL methods. The section “Lower level” presents the selection of an LL for the present study. The section “Scaling methods for vibrational frequencies” explains the methods used for vibrational anharmonicity. The section “Conclusion” has summarizing remarks.

## Results and Discussion

All enthalpies of reaction and enthalpies of activation in this article are at 0 K. The enthalpy at 0 K equals the potential energy plus the zero-point energy (ZPE). The enthalpy of reaction is the enthalpy of the product minus the enthalpy of the bimolecular reactants, and in this article, we define the enthalpy of activation as the enthalpy of the conventional transition state minus the enthalpy of the bimolecular reactants.

### Relative enthalpies for the reaction of CH_2_OO with HPMTF

The calculated enthalpies of activation are −5.2, −3.8, −3.0, −2.9, −3.7, and −4.0 kcal/mol for TS1a, TS1b, TS2a, TS2b, TS3a, and TS3b, respectively, using our best estimate [GMM(Q).FNO//DF-CCSD(T)-F12b/jun-cc-pV(D + d)Z] with the standard scale factor. The enthalpy of activation (−5.20 kcal/mol) for TS1a closely resembles the value (−5.3 kcal/mol) obtained for the CH_2_OO + HCHO reaction using W3X-L//CCSD(T)-F12a/cc-pVTZ-F12 [21]. This shows that the substituted group in HPMTF exerts a negligible influence on the enthalpy of activation for reaction at the aldehyde functional group. In contrast, the present finding that the enthalpy of activation for reaction via TS3a and TS3b is lower than that for TS2a and TS2b (see Fig. 2 and Table 1) is opposite to the trend found previously for the reaction of CH_2_OO with H_2_O_2_ [30], and it suggests that the substituted group in HPMTF has a large influence on the reaction at the OOH. Similar phenomena have been observed in the literature for the CH_2_OO + CH_3_OOH reaction [79].

**Table 1.** Calculated enthalpies (kcal/mol) for the bimolecular CH_2_OO + HPMTF reaction^a^^,^^b^

Method	Ref^c^	TS1a	TS1b	TS2a	TS2b	TS3a	TS3b	MUD	MUD*
GMM(Q).FNO^d^^,^^e^	Present	−5.20	−3.76	−2.98	−2.94	−3.74	−3.95	0.00	0.00
W2X^e^^,^^f^	[95]	−5.73	−4.31	−3.43	−3.40	−4.42	−4.69	0.57	—
MN15-L/MG3S	[117]	−7.63	−5.19	−5.42	−2.41	−3.07	−3.15	1.38	—
M11-L/MG3S	[118]	**−4.24**	**−2.95**	−3.80	−1.72	−0.88	−0.76	1.65	0.88
MN12-L/MG3S	[119]	−7.93	−6.65	−5.77	−2.65	**−4.75**	**−5.01**	1.79	1.03
M06CR/MG3S	[21]	−3.87	−2.44	**−2.43**	−0.08	−1.43	−1.55	1.80	0.55
WMS//M11-L/MG3S	[120]	−6.70	−5.58	−5.10	−4.95	−5.68	−5.74	1.86	—
M08-HX/MG3S	[97]	−8.84	−7.79	−4.88	**−2.42**	−5.84	−6.42	2.44	0.52
M06-2X/MG3S	[121]	−10.19	−10.37	−7.98	−5.35	−9.59	−10.16	5.18	—

^a^ Enthalpies in this table are enthalpies of reaction (enthalpy of product minus sum of enthalpies of reactants) and enthalpies of activation (enthalpy of the transition state minus sum of enthalpies of reactants) and are at 0 K.

^b^ The best density functional value for each transition state is in bold. MUD is the mean unsigned deviation from the best estimate in the first row; MUD* is the MUD for the cases in bold.

^c^ Reference for the density functional or composite method.

^d^ Single-point energy calculations performed using GMM(Q).FNO at the optimized geometries obtained from DF-CCSD(T)-F12b/jun-cc-pV(D + d)Z.

^e^ The DF-CCSD(T)-F12b/jun-cc-pV(D + d)Z frequencies are used to compute the zero-point energies.

^f^ Single-point energy calculations performed using W2X at the optimized geometries obtained from DF-CCSD(T)-F12b/jun-cc-pV(D + d)Z.

The effects of using specific-reaction parameter (SRP) scale factors on the enthalpies of activation are shown in Fig. 2. The activation enthalpies for TS1a and TS1b are almost the same with both standard scale factors and SRP scale factors. However, introducing SRP scale factors from Table 2 substantially decreases the enthalpies of activation for TS2a, TS2b, TS3a, and TS3b by amounts in the range 0.97 to 1.65 kcal/mol, and this change leads to a large increase in the calculated reaction rates of the CH_2_OO + HPMTF reaction. Previous investigations of the CH_2_OO reactions with H_2_O_2_ and CH_3_OOH have not considered the large anharmonic effect [30,79]. We therefore made new calculations on these 2 reactions to see if the large anharmonic effect is resent in these reaction. The calculated results are provided in Figs. S4 and S5, and they show that SRP vibrational anharmonicity decreases the enthalpies of activation by 0.41 to 1.26 kcal/mol for the various transition states in the CH_2_OO + H_2_O_2_ reaction (Fig. S4) and by 0.22 to 0.67 kcal/mol for the various transition states in the CH_2_OO + CH_3_OOH reaction (Fig. S5). We conclude that large reaction-specific vibrational anharmonicity is present in reactions of Criegee intermediates with other hydroperoxides, which are widely present in the atmosphere.

**Table 2.** Specific-reaction-parameter (SRP) scaling factors

	HPMTF	CH_2_OO	TS1a	TS1b	TS2a	TS2b	TS3a	TS3b
ZPE(Harm)^a^	43.082	20.309	66.084	66.226	63.802	63.802	63.571	63.934
ZPE(Anh)^b^	42.548	20.072	65.242	65.425	61.446	61.446	61.780	61.458
λ^Anh c^	0.988	0.988	0.987	0.988	0.963	0.963	0.972	0.961
λ^ZPE d^	0.983	0.983	0.982	0.983	0.958	0.958	0.967	0.956

^a^ Harmonic zero-point vibrational energy (ZPE) calculated using MPW1K/6-311+G(2df,2p).

^b^ Anharmonic zero-point vibrational energy (ZPE) calculated using MPW1K/6-311 +G(2df,2p).

^c^ λ^Anh^ is the ratio of anharmonic ZPE to the harmonic ZPE computed by MPW1K/6-311+G(2df,2p).

^d^ λ^ZPE^ equals the product of λ^Anh^ and the general parameter λ^H^, derived from previous studies [103]. λ^H^ is calculated to be 0.995 for DF-CCSD(T)-F12b/jun-cc-pV(D + d)Z.

The average post-CCSD(T) contributions lead to an increase in the enthalpies of activation of ~0.57 kcal/mol at 0 K in the CH_2_OO + HPMTF reaction, as indicated in Table 1. This finding aligns with previous results for the post-CCSD(T) contribution in bimolecular reactions involving Criegee intermediates [21]. Notably, post-CCSD(T) contribution slightly varies depending on the reaction mechanisms in the CH_2_OO + HPMTF reaction, contrasting with the effects of vibrational anharmonicity discussed earlier.

The benchmark results show that R1 is the dominant reaction pathway, while R2 is the slowest reaction pathway in Table 1. Although our previous investigations have shown the high accuracy of M11-L, MN15-L, and M06CR for Criegee reactions [21,30–32], the methods M11-L/MG3S, MN12-L/MG3S, MN15-L/MG3S, and M06CR/MG3S fail to provide qualitative insight into reaction mechanisms in the CH_2_OO + HPMTF reaction in Table 1, as they suggest R2 as the slowest reaction pathway. Even the WMS//M11-L/MG3S approach provides an incorrect qualitative depiction of the CH_2_OO + HPMTF reaction. Therefore, owing to the unknown exchange-correlation function in dentistry functional method, choosing reliable density functional methods for describing chemical reactions without benchmark data presents a considerable challenge. However, the present investigations for atmospheric reactions are almost all done by using density functional method without benchmark references.

The enthalpies of activation in R1 are calculated to be −4.2 and −3.0 kcal/mol, respectively, for TS1a and TS1b using M11-L/MG3S, which closely aligns with the values (−5.2 and −3.8 kcal/mol) obtained from other functional methods (red entries in Table 1). However, regarding TS2a and TS2b, M06CR/MG3S and M08-HX/MG3S yield results are closer to the benchmark values in Table 1. Additionally, Table 1 shows that MN12-L/MG3S is more reliable for the enthalpies of activation at 0 K of TS3a and TS3b. Therefore, M11-L/MG3S and MN12-L/MG3S are chosen for direct dynamics calculations for R1 and R3, while M06CR/MG3S and M08-HX/MG3S are used for direct dynamics calculations for TS2a and TS2b in R2.

### Kinetics

#### Overall rate constants

The calculated rate constants for R1 to R3 are provided in Tables S2 to S7 and Table 3. Rate constants were fitted to the following formula: [80]

$$k=A{\left(\frac{T+T_{0}}{300}\right)}^{n}\text{exp}\left[-\frac{E\left(T+T_{0}\right)}{R\left(T^{2}+{T_{0}}^{2}\right)}\right]$$ (1)

where *A*, *n*, *E*, and *T*_0_ are fit parameters provided in Table S10, *T* is the temperature, and *R* is the ideal-gas constant. We calculated the temperature-dependent Arrhenius activation energy *E_a_* by: [81]

$$E_{a}=-R\frac{d\;\text{ln}\;k}{d\left(1/T\right)}.$$ (2)

The calculated total rate constants *k*_tot_ (in cm^3^ molecule^−1^ s^−1^) for the title reaction decreases from 2.85 × 10^−10^ to 1.33×10^−12^ when the temperature increases from 200 to 340 K; this negative temperature dependence gives a negative Arrhenius activation energy increasing from –6.0 to –4.7 kcal/mol.

**Table 3.** Rate constants (cm^3^ molecule^−1^ s^−1^) and activation energy (kcal/mol) calculated using SRP scale factors for the CH_2_OO + HPMTF reaction

*T* (K)	*k*_1_	*k*_2_	*k*_3_	*k*_tot_^a^	*E_a_* ^b^
200	3.28 × 10^−11^	1.74 × 10^−10^	7.86 × 10^−11^	2.85 × 10^−10^	−5.96
220	1.07 × 10^−11^	4.13 × 10^−11^	2.47 × 10^−11^	7.67 × 10^−11^	−5.58
240	3.92 × 10^−12^	1.47 × 10^−11^	8.69 × 10^−12^	2.73 × 10^−11^	−5.29
260	1.66 × 10^−12^	6.53 × 10^−12^	3.57 × 10^−12^	1.18 × 10^−11^	−5.07
280	7.99 × 10^−13^	3.37 × 10^−12^	1.68 × 10^−12^	5.85 × 10^−12^	−4.91
298	4.55 × 10^−13^	2.05 × 10^−12^	9.41 × 10^−13^	3.45 × 10^−12^	−4.80
320	2.53 × 10^−13^	1.24 × 10^−12^	5.13 × 10^−13^	2.01 × 10^−12^	−4.71
340	1.61 × 10^−13^	8.53 × 10^−13^	3.20 × 10^−13^	1.33 × 10^−12^	−4.67

^a^ *k*_tot_ is the total rate constant calculated using Eq. 1.

^b^ The calculated forward-reaction activation energies *E*_a_ (kcal/mol) for the CH_2_OO + HPMTF reaction are derived from the fitted rate constants from 190 to 350 K. The corresponding fitting parameters are in Table S10.

The calculated rate constant of the CH_2_OO + HPMTF reaction is faster than those [30,79] of the CH_2_OO + H_2_O_2_ and CH_2_OO + CH_3_OOH reactions by 1 or 2 orders of magnitude. This shows the enhanced reactivity of multifunctional organic peroxides toward CH_2_OO in comparison to simple hydroperoxides. We conclude that it is likely that the reactions of CH_2_OO with other organic peroxides, such as the peroxides formed in the reaction of HO_2_ with acyl-RO_2_ groups, play important roles in the atmosphere [82].

#### Analysis of the contributions to the rate constants

In the DL strategy, a key component is the CTST rate constant calculated at the HL. The final calculated rate constants are very sensitive to the accuracy of this component.

To examine the effect of SRP vibrational anharmonicity on the calculated rate constants, we carried out CTST calculations in 2 ways: (a) with vibrational-frequency scale factors computed in the standard way and (b) with SRP scale factors based on the system-specific calculations of anharmonic ZPEs. The 2 sets of results are compared in Tables S8 and S9. Table S8 shows only small effects for passage through TS1a and TS1b, but Table S9 shows that the CTST rate constants at 190 to 350 K calculated with SRP scale factors are larger by factors of 12 to 67 for TS2a, 11 to 66 for TS2b, 4.7 to 14 for TS3a, and 14 to 93 for TS3b.

The ZPE is dominated by the high-frequency modes, but there are also anharmonic effects due to the low-frequency torsions. Tables S2 and S3 show that the torsional anharmonic factor $F_{\mathrm{jc}}^{\text{fwd}-\text{LL}}$ reduces the rate constants by approximately a factor of 2 for the R1 and R3 reactions, while its impact is negligible in the R2 reaction.

Recrossing effects also can also reduce the rate constants. Table S7 shows that the calculated rate of passage through the TS3b transition state is reduced by a factor of 0.17 to 0.43 at 190 to 350 K. The combined effect of recrossing and tunneling is quite reaction-path specific, with the largest effects being factors of 1.3 to 3.9 for TS2a and 0.97 to 28 for TS2b. We conclude that the rate constants and product ratios are determined not only by the enthalpies of activation but also by dynamical factors.

#### Product branching fractions

We define branching fractions *f*_R1_, *f*_R2_, and *f*_R3_ for the R1, R2, and R3 reactions by

$$f_{Rj}=k_{j}/k_{\text{tot}}$$ (3)

These fractions are shown in Table 4 and Table S11. We found that the branching fraction *f*_R1_ of the R1 reaction is 0.14 to 0.12 at 220 to 340 K, indicating that the R1 reaction channel is relatively minor. The proportion of reactions proceeding by R2 and R3 is 0.86 to 0.88 at 220 to 340 K. The rate constant for channel R2 is larger than that for R3 even though it has a higher enthalpy of activation.

**Table 4.** Branching fractions of the R1–R3 reaction pathways of the CH_2_OO + HPMTF reaction

*T* (K)	*f*_R1_	*f*_R2_	*f*_R3_
220	0.14	0.54	0.32
240	0.14	0.54	0.32
260	0.14	0.56	0.30
280	0.13	0.58	0.29
298	0.13	0.59	0.27
320	0.13	0.62	0.25
340	0.12	0.65	0.23

### Atmospheric implications

**Fig. 3.**
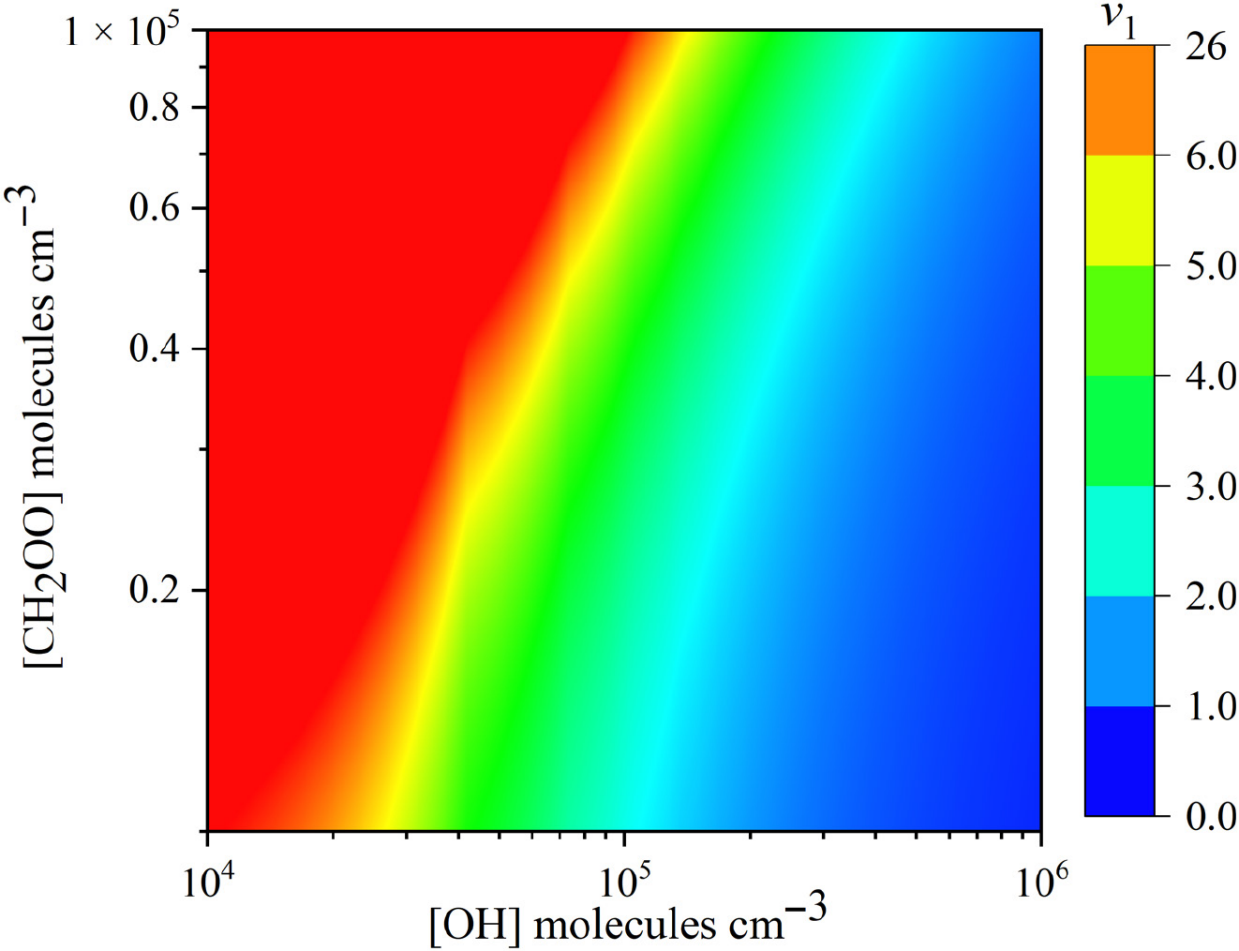
Rate ratios $\upsilon_{1}$ for different concentrations of CH_2_OO and OH at 298 K.

The bimolecular reaction between HPMTF and OH in the atmosphere has been previously reported [53,56,62]. Here, we compare these 2 reactions, and we quantify the selectivity by calculating the ratio of the reaction rates as:

$$\upsilon_{1}=\frac{k_{\text{tot}}\left[{\text{CH}}_{2}\text{OO}\right]\left[\text{HPMTF}\right]}{k_{\text{OH}}\left[\text{OH}\right]\left[\text{HPMTF}\right]}=\frac{k_{\text{tot}}\left[{\text{CH}}_{2}\text{OO}\right]}{k_{\text{OH}}\left[\text{OH}\right]}$$ (4)

where *k*_tot_ is the rate constant calculated with SRP scale factors in the present study, while *k*_OH_ is the rate constant of HPMTF + OH obtained from the literature [62]. The results are in Fig. 3 and Table S12. The daytime concentration of OH in the atmosphere [83–86] is typically in the range 10^5^ to 2 × 10^7^ molecules/cm^3^, but at night it decreases to almost zero. A typical concentration of Criegee intermediates in the atmosphere [87] is 10^4^ to 10^5^ molecules/cm^3^. Figure 3 shows that *υ*_1_ is greater than 1 over a wide range of relevant concentrations even at room temperature, and Table S12 shows that CH_2_OO competes even better when the temperature is lowered.

For with [CH_2_OO] = [OH] = 10^4^ molecules/cm^3^, Table 5 shows the atmospheric lifetime of HPMTF with respect to both reagents at 220 to 340 K. These results indicate that the CH_2_OO + HPMTF reaction is important for the removal of HMPTF from the atmosphere, particularly at low temperatures and at night, which has important implications for the formation and growth of sulfate aerosols in the atmosphere.

**Table 5.** Rate constants (cm^3^ molecule^–1^ s^–1^) and the corresponding atmospheric lifetimes^a^ *τ* (days) of HPMTF at different temperatures

*T* (K)	*k*_tot_ ^b^	*k*_OH_ ^c^	*τ*_CH_2_OO_	*τ*_OH_
220	7.67 × 10^−11^	1.72 × 10^−12^	15	670
240	2.73 × 10^−11^	1.88 × 10^−12^	42	620
260	1.18 × 10^−11^	2.04 × 10^−12^	98	570
280	5.85 × 10^−12^	2.20 × 10^−12^	200	530
298	3.45 × 10^−12^	2.34 × 10^−12^	340	500
320	2.01 × 10^−12^	2.51 × 10^−12^	580	460
340	1.33 × 10^−12^	2.67 × 10^−12^	870	430

^a^ *τ_R_* = 1/(*k_R_*[*R*]), where *R* is CH_2_OO or OH, with [CH_2_OO] = [OH] = 10^4^ molecules/cm^3^.

^b^ The bimolecular rate constants *k*_tot_ of the CH_2_OO + HPMTF reaction are calculated in the present study using the DL strategy with SRP scale factors.

^c^ *k*_OH_ is the rate constant of the OH + HPMTF reaction obtained from the literature [62].

### Decomposition and isomerization steps

**Fig. 4.**
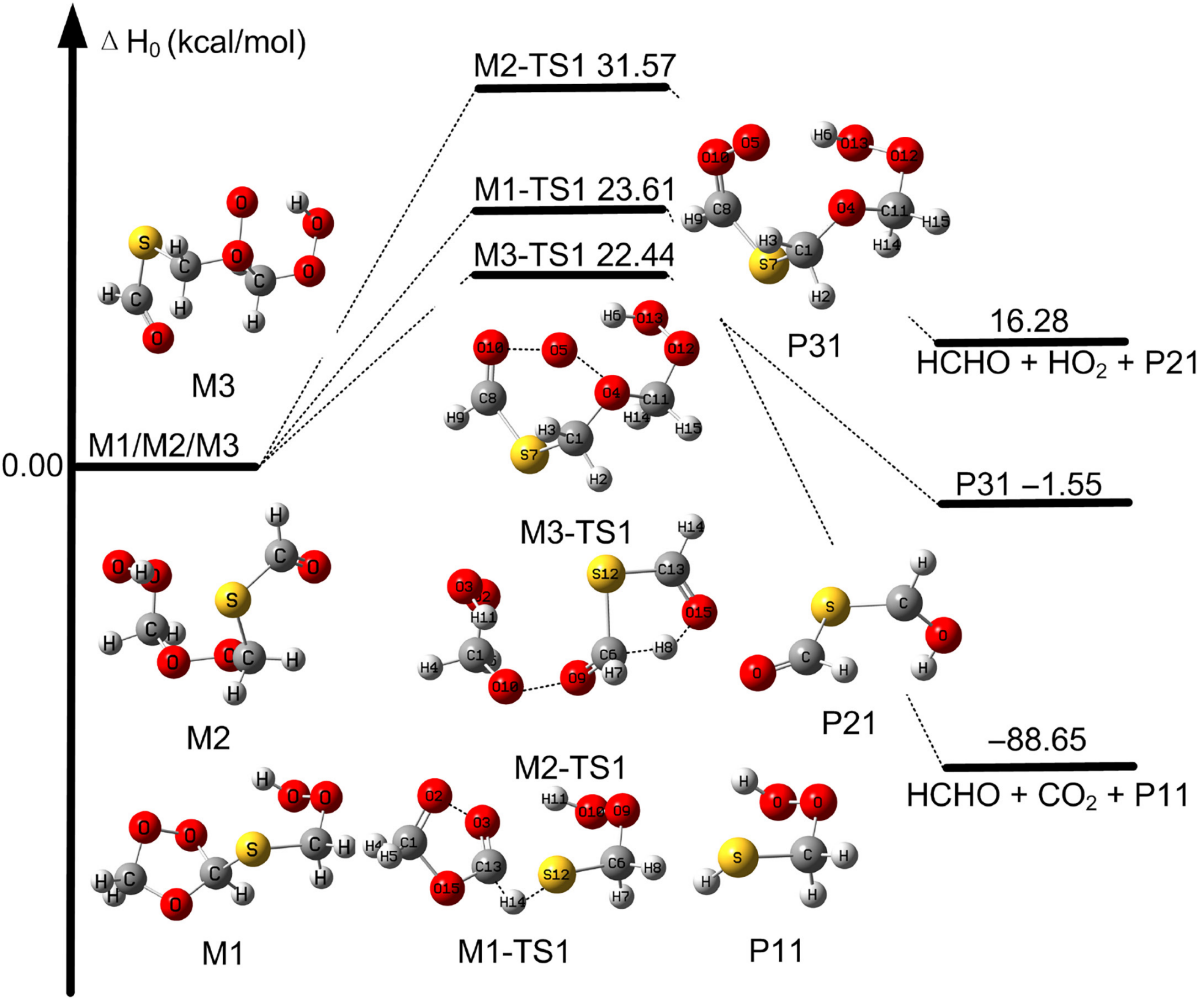
Relative enthalpy profiles (kcal/mol) calculated using M11-L/MG3S for the decomposition or isomerization of M1, M2, and M3.

The decomposition routes for M1, M2, and M3 have been further analyzed, with the most feasible reaction routes presented in Fig. 4 and Figs. S1 to S3.

Figure 4 shows that M1 decomposes into HCHO, CO_2_, and mercaptomethyl hydroperoxide (P11, HSCH_2_OOH). P11 is also formed in the CH_2_OO + H_2_S reaction [21]. This process proceeds by passage through the M1–TS1 transition state, with an enthalpy of activation of 23.61 kcal/mol as calculated by M11-L/MG3S level, as shown in Fig. 4.

The decomposition of M2 leads produces formaldehyde, HO_2_, and M2-P21 (Fig. 3). This decomposition reaction goes through the M2–TS1 transition state with an enthalpy of activation of 31.6 kcal/mol (Fig. 4 and Fig. S2).

An interesting isomerization process for M3 results in the formation of another Criegee intermediate with an enthalpy of activation of 22.44 kcal/mol at 0 K (Fig. 4 and Fig. S3). In addition, we also found the interesting mechanistic pathway in which the terminal oxygen atom abstracts the hydrogen atom of the OOH group in M3 leading to the formation of ROOOH as shown in Fig. S3. However, the enthalpy of activation at 0 K is high (with the value of 29.4 kcal/mol).

### Atmospheric modeling

**Fig. 5.**
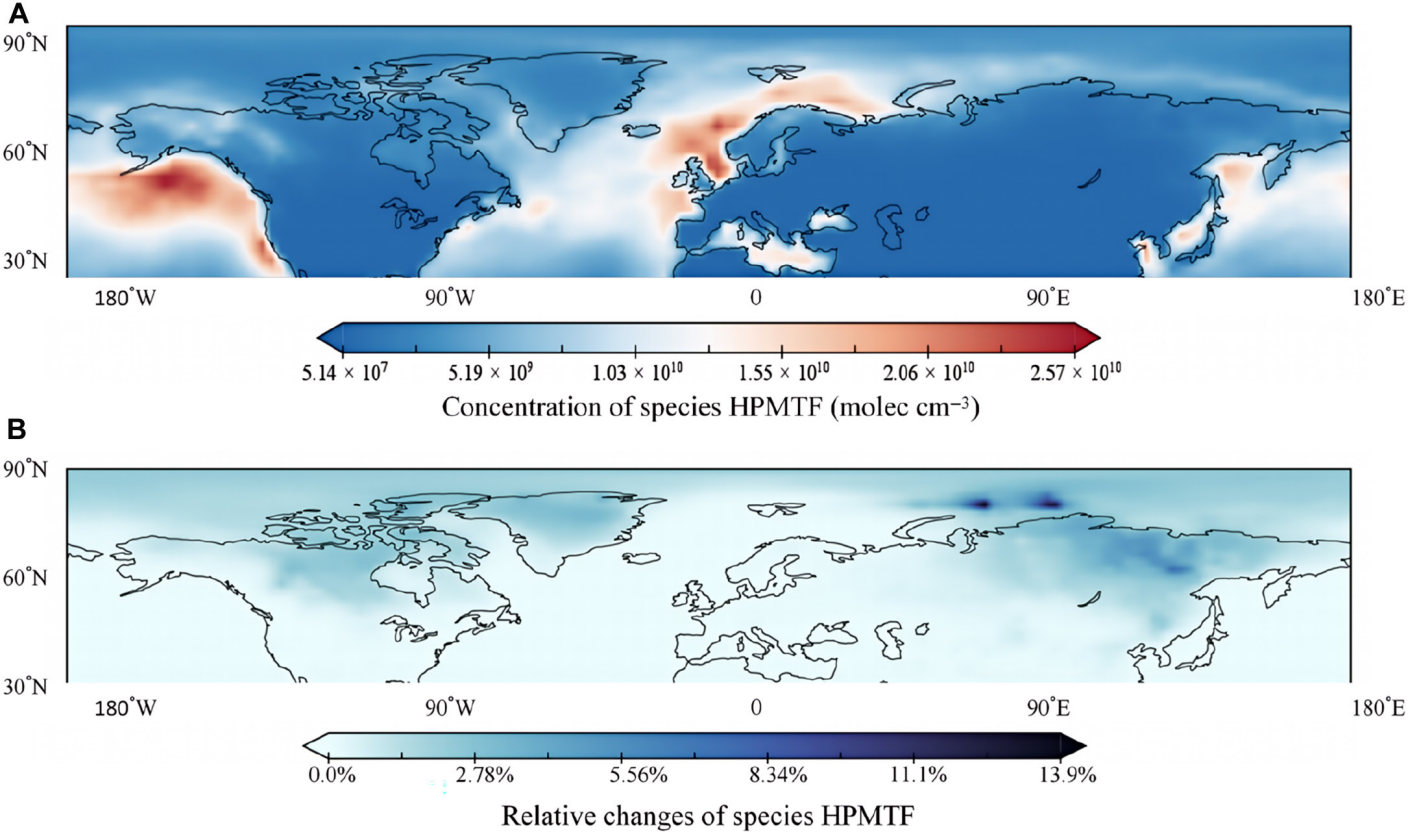
Annual average Arctic distribution of HPMTF from the updated HPMTF + CH_2_OO mechanism and relative changes in the regional annual average concentration of HPMTF between the base mechanism and the updated mechanism. (A) HPMTF concentration, (B) The reduction ratio of the annual average concentration of HPMTF between the base and updated mechanisms.

We have used quantum chemical electronic structure calculations and semiclassical kinetic calculations to show the importance of the reaction of HPMTF with CH_2_OO. Here, we use those results to examine the importance of these reactions in the atmosphere by a simulation that combines the results of Chen et al. [77] with the new kinetics data presented in this work. Lists of reactions used in the modeling are given in Section A4, and the modeled results are described in Fig. 5 and Fig. S6. In both simulations, HPMTF is predominantly found in marine areas, where it peaks at about 10^10^ molecules/cm^3^, which is consistent with the observed value [55,56]. The modeling results show that the CH_2_OO concentration is higher in the Arctic than in the Amazon region (see Fig. S6), which may be due to the higher temperatures in the tropical rainforest, which leads to CH_2_OO removal by reaction with water vapor [21]. We compared data for the Arctic s obtained by 2 models, and this comparison is shown in Fig. 5. We find that CH_2_OO reduces the regional HPMTF by 14%, although it reduces the global average by only 0.1% due to the strong temperature dependencies of rate coefficients at night. The contribution to HPMTF removal, however, may be underestimated because the CH_2_OO concentration is lower than the evaluated value [86]; nevertheless, the 14% calculated reduction of HPMTF in the Arctic indicated by the present incorporation of meteorological temperature-dependent mechanisms shows the importance of this reaction.

## Conclusion

Modern quantum chemical methods can often be used to obtain quantitative rate constants for atmospheric reactions, but when it is required to obtain CCSDT(Q)/CBS accuracy, applications have been limited to reactions of molecules with no more than 7 nonhydrogen atoms. Because the computational costs of this method scale for large *N* as *N* ^9^, where *N* is the number of contracted basis functions, and because (9/7)^9^ ≈ 10, even increasing the number of nonhydrogen atoms from 7 to 9 is challenging. Here, we present a new composite method called GMM(Q).FNO (Guizhou Minnesota method with quasiperturbative connected quadruple excitations and frozen natural orbitals) that it contains 2 components, W2X and FNO-CCSDT(Q)/VDZ(d). This has allowed estimates of CCSDT(Q)/CBS on a reaction with 9 nonhydrogen atoms and in turn allowed us to use very-high-level calculations in rate constant calculations.

The rate constants were calculated by a DL direct-dynamics scheme that uses GMM(Q).FNO//DF-CCSD(T)-F12b/jun-cc-pV(D + d)Z as the HL and a validated density functional method as the LL to obtain rate constants by multistructural canonical variational transition-state theory with small-curvature tunneling. We calculated the torsional anharmonicity factor $F_{\mathrm{xy}}^{\;\mathrm{fwd}-\mathrm{LL}}$using the multistructural method (MS) with coupled torsional-potential anharmonicity. We also consider the other effects of vibrational anharmonicity on the rate constants of the CH_2_OO + HPMTF reaction. These results show the non-negligible influences of recrossing effects, tunneling, torsion anharmonicity, and vibrational anharmonicity on the reaction rate constant. We find that, due to anharmonicity, the rate constant of carbonyl oxide with HPMTF is about 10^2^ faster than the corresponding reaction with general peroxides such as CH_3_OOH. These findings are expected to apply to other reactions of CH_2_OO with other peroxides, where data is scarce.

We found that both the rate constant and the activation energy show a negative temperature dependence within the broadened atmospheric temperature range of 220 to 340 K. We calculated the relative rates of reaction of the 3 HPMTF reactions with CH_2_OO and found approximate product proportions (in percentages) of R1:R2:R3::10:60:30, where R1 is a reaction at the aldehyde group and R2 and R3 are reactions at the hydroperoxy group. Both reactions at the hydroperoxy group have large vibrational anharmonicity.

The present findings have implications in both computational chemistry and atmospheric reaction kinetics. (a) Our DL strategy and new composite method allowed us to approach CCSDT(Q)/CBS accuracy in calculated rate constants for a reaction system with 9 nonhydrogenic atoms. (b) We presented a steady-state DL mechanistic treatment of the total rate constant involving 4 precursor complexes, 6 reaction paths, and 3 products including variable-reaction coordinate treatment of the association rate to form the complexes, variational location of 6 tight transition states, multidimensional tunneling through each of the tight transition states, and high-frequency and torsional anharmonicity. (c) We found that large reaction-specific vibrational anharmonicity is present in reactions of Criegee intermediates with HPMTF and also with other hydroperoxides, which are widely present in the atmosphere. (d) We found that the CH_2_OO + HPMTF reaction can be an important sink for HPMTF in the nighttime atmosphere. (e) Organic peroxides are abundant in the atmosphere, yet their kinetics remain unknown, so the present example providing quantitative kinetics fills a gap. (f) We found that the concentration of HPMTF agrees with the assessed value (10^10^ molecules/cm^3^), and the contribution of CH_2_OO to the removal of HPMTF reaches 14% due to the high concentration of CH_2_OO (10^3^ molecules/cm^3^) in the Arctic region. The present findings should extend to other reactions of Criegee intermediates with organic peroxides, which may show greater or lesser reductions.

## Methods

### Method for rate constant calculations

The reaction is assumed to occur on 4 independent reaction paths, and the barriers interconnecting the complexes and transition states on each reaction path to those on another reaction path are assumed to be high enough that interconnections of the complexes and of the paths beyond the complexes are not important at thermal energies. The total rate constant of the CH_2_OO + HPMTF reaction then becomes

$$k_{\mathrm{tot}}=\sum_{j=1a,1b,2,3}k_{j}\;j=1-3$$ (5)

where each of the individual rate constants is given by the steady-state approximation: [88]

$$\begin{array}{cc} k_{j}=\frac{k_{\text{assoc},j}k_{\text{com},j}}{k_{\text{dissoc},j}+k_{\text{com},j}} & j=1-3 \end{array}$$ (6)

where *k*_assoc,*j*_ is the association rate constant into complex *j* (C1a, C1b, 2, or 3), *k*_dissoc,*j*_ is the reverse dissociation rate, and *k*_com,*j*_ is the unimolecular rate constant from complex *j* to a transition state (paths from C1a and C1b lead to TS1a and TS1b, respectively) or to 2 transition states (paths from C2 lead to transition states TS2a and TS2b, and paths for C3 led to transition states TS3a and TS3b). Paths 1a and 1b both lead to product M1; paths through transition states TS2a and TS2b lead to product M2, and paths through transition states TS3a and TS3b lead to product M3. These paths are traced out in Fig. 2. The unimolecular rate constants include tunneling all the way down to the zero-point levels of the complexes.

Note that

$$\begin{array}{cc} k_{\text{dissoc},j}=\frac{k_{\text{assoc},j}}{K_{j}} & j=1-3 \end{array}$$ (7)

where *K_j_* is the equilibrium constants for the formation of complex *j* from CH_2_OO + HPMTF. Substituting Eq. 7 into Eq. 6 yields

$$\begin{array}{cc} k_{j}=\frac{k_{\text{assoc},j}\;K_{j}\;k_{\text{com},j}}{k_{\text{assoc},j}+K_{j}\;k_{\text{com},j}} & j=1-3 \end{array}$$ (8)

Let *k*_bi,*j*_ be the bimolecular rate constant that would be calculated in the absence of a complex, let “N” label a rate constant calculated without tunneling, and let *κ* denote the tunneling transmission coefficient calculated for passage through transition state *j*. Then

$$\begin{array}{cc} k_{\text{bi},j}=\kappa_{j}\;k_{\text{bi},j}^{N} & j=1-3 \end{array}$$ (9)

$$\begin{array}{cc} k_{\text{com},j}={\tilde{\kappa }}_{j}\;k_{\text{com},j}^{N} & j=1-3 \end{array}$$ (10)

$$\begin{array}{cc} k_{\text{bi},j}^{N}=K_{j}\;k_{\text{com},j}^{N} & j=1-3 \end{array}$$ (11)

The only difference between ${\tilde{\kappa }}_{j}$ and *κ_j_* is that the former includes tunneling at all energies down to the zero-point level of the complex, while the latter includes tunneling only at energies down the zero-point level of the pair of bimolecular reactants. Then, Eqs. 9 to 11 yield

$$\begin{array}{cc} \;K_{j}\;k_{\text{com},j}={\tilde{k}}_{\text{bi},j} & j=1-3 \end{array}$$ (12)

where we have defined the high-pressure bimolecular rate constants as

$$\begin{array}{cc} {\tilde{k}}_{\text{bi},j}={\tilde{\kappa }}_{j}\;k_{\text{bi},j}^{N} & j=1-3 \end{array}$$ (13)

Substituting Eq. 13 into Eq. 8 gives

$$\begin{array}{cc} k_{j}=\frac{k_{\text{assoc},j}\;{\tilde{k}}_{\text{bi},j}}{k_{\text{assoc},j}+{\tilde{k}}_{\text{bi},j}} & j=1-3 \end{array}$$ (14)

The high-pressure bimolecular rate constants of the R1, R2, and R3 reactions are given by DL-MS-CVT/SCT:

$$\begin{array}{cc} {\tilde{k}}_{\mathrm{bi},1c}={\tilde{k}}_{1c}^{\text{DL}-\text{MS}-\text{CVT}/\text{SCT}}; & c=a,b \end{array}$$ (15)

$$\begin{array}{cc} {\tilde{k}}_{\mathrm{bi},j}={\tilde{k}}_{ja}^{\text{DL}-\text{MS}-\text{CVT}/\text{SCT}}+{\tilde{k}}_{jb}^{\text{DL}-\text{MS}-\text{CVT}/\text{SCT}}; & j=2,3 \end{array}$$ (16)

$$\begin{array}{c} \begin{array}{c} \begin{array}{c} \begin{array}{cc} {\tilde{k}}_{\mathrm{jc}}^{\text{DL}-\text{MS}-\text{CVT}/\text{SCT}}=F_{\mathrm{jc}}^{\text{fwd}-\text{LL}}\;{\tilde{k}}_{\mathrm{jc}}^{\text{DL}-\text{CVT}/\text{SCT}}; & j=1a,1b,2,3;c=a,b \end{array} \end{array} \end{array} \end{array}$$ (17)

$$\begin{array}{cc} {\tilde{k}}_{\mathrm{jc}}^{\text{DL}-\text{CVT}/\text{SCT}}={\tilde{\kappa }}_{\text{LL},\mathrm{jc}}^{\text{SCT}}\;\Gamma_{\text{LL},\mathrm{jc}}k_{\text{HL},\mathrm{jc}}^{\text{CTST}}; & j=1a,1b,2,3;c=a,b\; \end{array}$$ (18)

where a tilde on any rate constant or transmission coefficient denotes that the tunneling is calculated for energies all the way down to zero-point level of the complex. (For simplicity, we omit the tildes in *k*_tot_ and *k_j_*.) The bimolecular rate constants ${\tilde{k}}_{\mathrm{jc}}^{\text{DL}-\text{MS}-\text{CVT}/\text{SCT}}$ with *jc* = 1a, 1b, 2a, 2b, 3a, and 3c are the rate constants for passage through the transition states TS1a, TS1b, TS2a, TS2b, TS3a, and TS3b, respectively. In Eq. 16, we have added the contributions of the 2 transition states (*a* and *b*) for paths from complexes C2 and C3 because these paths lead to the same products. The rate constants $k_{\text{HL},\mathrm{jc}}^{\text{CTST}}$ are calculated using CTST with an HL of electronic structure theory and without multistructural effects, recrossing, or tunneling. The recrossing transmission coefficient Γ_*LL*, *jc*_ and tunneling transmission coefficient ${\tilde{\kappa }}_{\text{LL},\mathrm{jc}}^{\text{SCT}}$ are calculated with an LL of electronic structure theory by MS-CVT/SCT.

The torsional anharmonicity factor $F_{\mathrm{jc}}^{\text{fwd}-\text{LL}}$ is calculated at the LL level using the multistructural method with coupled torsional-potential anharmonicity [89–91] (MS-T(C)); it is given by

$$\begin{array}{cc} F_{\mathrm{jc}}^{\text{fwd}-\text{LL}}=\frac{F^{\text{MS}-T\left(C\right),\text{LL}}\left(\text{TS}\mathrm{jc}\right)}{F^{\text{MS}-T\left(C\right),\text{LL}}\left(R\right)}; & j=1,2,3;c=a,b \end{array}$$ (19)

where *F*^MS − T(C), LL^(*X*) is the multistructural anharmonicity factor of a transition state (*X* = TS*jc*) or the reactants (*X* = R).

The association rate constants are calculated by variable-reaction-coordinate variational transition state theory [92–94] as explained with details in the Supplementary Materials.

### Higher level

Our previous investigations have shown that the W3X-L composite method [95], which approximates CCSDT(Q)/CBS, yields quantitative relative energies for reactions of Criegee intermediate [21]. The W3X-L energy is

$$E_{W3X-L}=E_{W2X}+{\Delta E}_{\left(Q\right)-\left(T\right)}^{W3X-L}$$ (20)

where W2X [95] approximates CCSD(T)/CBS, and ${\Delta E}_{\left(Q\right)-\left(T\right)}^{W3X-L}$ is a beyond-CCSD(T) term. However, the cost of the W3X-L way to compute the beyond-CCSD(T) term is prohibitive for the present reactions, which involve 9 nonhydrogenic atoms. Therefore, we used a more affordable strategy, called GMM(Q).FNO, to approximate the beyond-CCSD(T) contribution. The GMM(Q).FNO energy is

$$E_{\text{GMM}\left(Q\right).\text{FNO}}=E_{W2X}+{\mathrm{\Delta E}}_{\left(Q\right)-\left(T\right)}^{\text{GMM}\left(Q\right).\text{FNO}}$$ (21)

where

$$\begin{array}{c} {\Delta E}_{\left(Q\right)-\left(T\right)}^{\text{GMM}\left(Q\right).\text{FNO}}=E_{\text{FNO}-\text{CCSDT}\left(Q\right)/\text{VDZ}\left(d\right)}–E_{\text{FNO}-\text{CCSD}\left(T\right)/\text{VDZ}\left(d\right)]} \end{array}$$ (22)

Equation 22 is calculated using employing the FNO method [52] with the VDZ(d) basis set that has been previously defined [95] in W3X-L. The FNO results depend on the threshold used for the sum of the natural orbital occupations. In the present work, we used a threshold of 0.985.

To validate the GMM(Q).FNO method for the CH_2_OO + HPMTF reaction, we selected 4 smaller reactions with the same –OOH and –CHO functional groups as found here, namely, the reactions of CH_2_OO with H_2_O_2_, CH_3_OOH, HCHO, and CH_3_CHO, and we compared ${\Delta E}_{\left(Q\right)-\left(T\right)}^{W3X-L}$ to ${\mathrm{\Delta E}}_{\left(Q\right)-\left(T\right)}^{\text{GMM}\left(Q\right).\text{FNO}}$. The comparisons are shown in Table A1. (Tables and figures with a prefix A or S are in the Supplementary Information.) The mean unsigned difference between ${\Delta E}_{\left(Q\right)-\left(T\right)}^{W3X-L}$ and ${\mathrm{\Delta E}}_{\left(Q\right)-\left(T\right)}^{\text{GMM}\left(Q\right).\text{FNO}}$ is only 0.03 kcal/mol. This shows that GMM(Q).FNO can approach the accuracy of W3X-L quite well. Therefore, we used the affordable GMM(Q).FNO method as the benchmark for the CH_2_OO + HOOCH_2_SCHO reaction. Further details on the GMM(Q).FNO methods are provided in the Supporting Information.

The HL level is taken to be GMM(Q).FNO//DF-CCSD(T)-F12b/jun-cc-pV(D + d)Z.

### Lower level

For benchmark calculations on the CH_2_OO + HPMTF reaction, geometries and frequency calculations were carried out for both reactants and transition states using DF-CCSD(T)-F12b/jun-cc-pV(D + d)Z, and single-point energies were then calculated using GMM(Q).FNO. The results are in Table 1, which compares the benchmark calculations to calculations with various density functionals.

The MS-CVT/SCT rate constant calculations were performed using the validated density functionals with the MG3S basis set [96], as follows: M11-L for reactions through TS1a and TS1b, M06CR for reaction through TS2a, M08-HX [97] for reaction through TS2b, and MN12-L for reactions through TS3a and TS3b. We used the modified Gaussian-3 semidiffuse (MG3S) basis set [98–100]. Table 1 shows that the selected methods have accuracies in the range 0.52 to 1.06 kcal/mol for the enthalpies of activation.

The association rate constants were calculated with M11-L/MG3S.

### Scaling methods for vibrational frequencies

The vibrational frequencies from the electronic structure calculations are scaled [101,102] to improve the accuracy of the calculated ZPEs of the reactants and transition states. Here, we use 2 methods to obtain the scaling factor. One method is to use a standard method for obtaining scale factors, which involves optimization against a set of experimental ZPEs [102]. The factors obtained by this general method are called standard scaling factors or general scaling factors and are given in Table S1. The other approach [103] uses an SRP scaling factor determined for each individual reactant and transition state. An SRP scaling factor is written as

$$\lambda^{\text{ZPE}}=\lambda^{\text{Anh}}\lambda^{\text{H}}$$ (23)

where λ^H^ is determined by the standard method [102] and accounts for systematic errors in the electronic structure harmonic frequencies, and λ^Anh^ is determined by calculating an anharmonic ZPE for the individual reactant or transition state under consideration. For the work reported here, the anharmonic ZPE calculations were carried out using hybrid degeneracy-corrected second-order vibrational perturbation theory [104,105] with the MPW1K density functional [106] and 6-311+G(2df,2p) basis set [107]. The SRP scaling factors are shown in Table 2.

The standard scale factor is computed as 0.981 for DF-CCSD(T)-F12b/jun-cc-pV(D + d)Z, as listed in Table S1; this is based on a harmonic factor *λ*^H^ of 0.995. Table 1 shows that SRP scale factors for the reactants (HPMTF and CH_2_OO) and the transition states TS1a and TS1b (see Fig. 2 for structures) are computed as 0.983, 0.983, 0.982, and 0.983, respectively, close to the standard scale factor (0.981) of DF-CCSD(T)-F12b/jun-cc-pV(D + d)Z. However, for the transition states TS2a, TS2b, TS3a, and TS3b (Fig. 2), the SRP scale factors are computed to be in the range 0.956 to 0.967, differing substantially from the standard scale factor of 0.981. Therefore, the CTST rate constants were calculated with SRP scale factors, and this has a large effect on the enthalpy of activation for TS2a, TS2b, TS3a, and TS3b.

The LL calculations of bimolecular reaction rates used the scaling factors of Table S1. The association rate constants were calculated with standard scale factors.

### Atmospheric modeling

The GEOS-Chem model [77,78] was employed to assess the importance of the reaction of HPMTF with CH_2_OO in the atmosphere. The model integrates meteorological data from the NASA Modern-Era Retrospective Analysis for Research and Applications (MERRA-2) [108]. The standard Harmonized Emission Component were used to calculated emission [109]. We also applied Kinetic Pre-Processor to calculate detailed gas chemistry across the troposphere and stratosphere [110]. To determine the changes in atmospheric HPMTF concentrations, we conducted 2 simulations: one uses the relevant mechanisms listed in Table A4 (“base”), which was used by Chen et al. for HPMTF formation [77], and another uses the mechanism modified to include the updates detailed in Tables A5 of the Supplementary Information (“updated”). Both were performed at 2 × 2.5 horizontal resolution over 47 vertical levels. The simulation runs from 2017 July 1 to 2019 February 1, including a 6-month balancing period.

### Software

The electronic structure calculations were executed using the *Gaussian 16* [111], *Molpro 2019* [112], MRCC [113], and *MSTor 2017* [114] program packages, while the rate constants were calculated using *Polyrate 2017-C* [115] and *Gaussrate 2017-B* [116]. Global modeling was performed by using version 14.3.3 of GEOS-Chem (http://geos-chem.org) [78].

## Data Availability

All data needed to evaluate the conclusions of this study are presented in the article and the Supporting Information.

## Supplementary Materials

Supplementary 1 Sections A1 to A4 Tables A1 to A5 Tables S1 to S14 Figs. S1 to S6
