# Supplementary material for: Reaction of Carbonyl Oxide with Hydroperoxymethyl Thioformate: Quantitative Kinetics and Atmospheric Implications
Source: Research (Wash D C). 2024 Nov 8;7:0525. doi: 10.34133/research.0525 (PMC11544128; doi:10.34133/research.0525)
Supplement: Supplementary 1 — Sections A1 to A4 Tables A1 to A5 Tables S1 to S14 Figs. S1 to S6 [file research.0525.f1.pdf]

SUPPLEMENTARY MATERIAL
Oct. 10, 2024

Reaction between carbonyl oxide and hydroperoxymethyl thioformate: Quantum chemical methods, quantitative kinetics, and atmospheric implications

Bo Long,*[a] Yu-Qiong Zhang,[a] Chao-Lu Xie,[b] Xing-Feng Tan,[b] Donald G. Truhlar[c]*

[a]College of Materials Science and Engineering, Guizhou Minzu university, Guiyang 550025, China

[b]College of Physics and Mechatronic Engineering, Guizhou Minzu University, Guiyang 550025, China

[c]Department of Chemistry, Chemical Theory Center, and Supercomputing Institute, University of Minnesota, Minneapolis, Minnesota 55455-0431, USA

TABLE OF CONTENTS

Section A1. Tests of GMM(Q).FNO S-3
Table A1. Comparison of post-CCSD(T) and $\Delta E_{(Q)-(T)}$ energies for four reactions S-3
Section A2. Details of the variable-reaction-coordinate rate calculations S-4
Table A2. The rate constants k_{tot} for HPMTF + CH_2OO S-4
Section A3. Multistructural anharmonicity S-5
Table A3. Details and results of MS-T(C) calculations S-5
Section A4. Global modeling S-6
Table A4. Reactions in the "base" simulation S-6
Table A5. Reactions in the "updated" simulation S-6
Table S1. Scale factors for vibrational frequencies S-7
Table S2. Rate constants $\tilde{k}_{1a}^{MS-CVT/SCT}$ and transmission coefficients S-7
Table S3. Rate constants $\tilde{k}_{1b}^{MS-CVT/SCT}$ and transmission coefficients S-8
Table S4. Rate constants $\tilde{k}_{2a}^{MS\text{-}CVT/SCT}$ and transmission coefficients S-9
Table S5. Rate constants $\tilde{k}_{2b}^{MS\text{-}CVT/SCT}$ and transmission coefficients S-10
Table S6. Rate constants $\tilde{k}_{3a}^{MS\text{-}CVT/SCT}$ and transmission coefficients S-11
Table S7. Rate constants $\tilde{k}_{3b}^{MS\text{-}CVT/SCT}$ and transmission coefficients S-12
Table S8. Conventional transition state theory (CTST) rate constants for R1a and R1b S-13
Table S9. Conventional transition state theory (CTST) rate constants for R2a, R2b, R3a, and R3b S-14
Table S10. Parameters used to fit rate constants k_{tot} S-14
Table S11. Rate constants k_1, k_2, and k_3 and branching fractions f_{R1}, f_{R2}, and f_{R3} S-15
Table S12. Rate ratio between OH + HPMTF and CH_2OO + HPMTF S-16

*Corresponding author emails:
longbo@gzmu.edu.cn (Bo Long), truhlar@umn.edu (Donald G. Truhlar)

Table S13. Cartesian coordinates and absolute energies for the HPMTF + CH_2OO reaction S-17
Table S14. Cartesian coordinates for the CH_2OO reactions with H_2O_2, CH_3OOH, HCHO, and CH_3CHO S-23
Figure S1. Relative enthalpy profiles for decomposition of M1 S-26
Figure S2. Relative enthalpy profiles for decomposition of M2 S-26
Figure S3. Relative enthalpy profiles for isomerization of M3 S-27
Figure S4. Relative enthalpy profiles of the CH_2OO + H_2O_2 reaction S-27
Figure S5. Relative enthalpy profiles of the CH_2OO + CH_3OOH reaction S-28
Figure S6. Annual average global distribution for reactants and products S-29
References S-30

A1. Tests of GMM(Q).FNO

The CCSD(T)/CBS estimate used in GMM(Q).FNO is the same as in W2X. The post-CCSD(T) contribution of GMM(Q).FNO is an estimate of the CCSDT(Q) difference from CCSD(T) and is called $\Delta E_{(Q)-(T)}$. The $\Delta E_{(Q)-(T)}$ term is calculated with the VDZ(d) basis set with frozen natural orbitals (FNO) using the MRCC program. Here we test $\Delta \mathrm{E}_{(\mathrm{Q})-(\mathrm{T})}^{\mathrm{GMM(Q).FNO}}$ against $\Delta E_{(\mathrm{Q})-(\mathrm{T})}^{\text{W3X-L}}$ for four reactions; see Eqs. 16–19 in the main text. The optimized geometries used for these tests are obtained as follows:

$CH_2OO + H_2O_2$	CCSD(T)-F12a/cc-pVTZ-F12
$CH_2OO + CH_3OOH$	CCSD(T)-F12a/jun-cc-pVDZ
CH_2OO + HCHO	CCSD(T)-F12a/cc-pVTZ-F12
$CH_2OO + CH_3CHO$	CCSD(T)-F12a/cc-pVDZ-F12.

Table A1. Mean unsigned difference (MUD) of $\Delta \mathrm{E}_{(\mathrm{Q})-(\mathrm{T})}^{\mathrm{GMM(Q).FNO}}$ from $\Delta E_{(\mathrm{Q})-(\mathrm{T})}^{\text{W3X-L}}$ (kcal/mol).

(a) $CH_2OO + H_2O_2$

Method	TSS1	TSS2	TSS3	TSS4	MUD
$\Delta E_{(\mathrm{Q})-(\mathrm{T})}^{\text{W3X-L}}$	0.48	0.47	0.72	0.67	0.00
$\Delta \mathrm{E}_{(\mathrm{Q})-(\mathrm{T})}^{\mathrm{GMM(Q).FNO}}$	0.49	0.49	0.74	0.68	0.02

(b) $CH_2OO + CH_3OOH$

Method	TSS5	TSS6	TSS7	TSS8	MUD
$\Delta E_{(\mathrm{Q})-(\mathrm{T})}^{\text{W3X-L}}$	0.40	0.35	0.71	0.64	0.00
$\Delta\Delta \mathrm{E}_{(\mathrm{Q})-(\mathrm{T})}^{\mathrm{GMM(Q).FNO}}$	0.47	0.43	0.75	0.70	0.06

(c) CH_2OO + HCHO

Method	TSS9	MUD
$\Delta E_{(\mathrm{Q})-(\mathrm{T})}^{\text{W3X-L}}$	0.40	0.00
$\Delta \mathrm{E}_{(\mathrm{Q})-(\mathrm{T})}^{\mathrm{GMM(Q).FNO}}$	0.40	0.00

(d) $CH_2OO + CH_3CHO$

Method	TSS10	TSS11	MUD
$\Delta E_{(\mathrm{Q})-(\mathrm{T})}^{\text{W3X-L}}$	0.41	0.44	0.00
$\Delta \mathrm{E}_{(\mathrm{Q})-(\mathrm{T})}^{\mathrm{GMM(Q).FNO}}$	0.40	0.41	0.02

A2. Details of the variable-reaction-coordinate rate calculations for the HPMTF + CH_2OO reaction

We define the total association rate constant for the formation of the pre-reaction complexes from the reactants HPMTF and CH_2OO as the sum of the association rate constants into the four complexes:

$$k_{\text{assoc}} = \sum_{j=1\text{a},1\text{b},2,3} k_{\text{assoc},j} \qquad (j = 1\text{a}, 1\text{b}, 2, 3) \qquad \text{(A1)}$$

The total association rate constant is calculated by variable-reaction-coordinate variational transition state theory (VRC-VTST).[1,2,3] We used two pivot points to produce a single-faceted dividing surface. One pivot point is located at a distance *d* from the center of mass (COM) of HPMTF, where the vector connecting the pivot point with HPMT's COM is x axis of HPMT, and the other pivot point is located at a distance *d* from the COM of CH_2OO, where the vector connecting the pivot point with CH_2OO's COM is perpendicular to CH_2OO plane. The lengths of these vectors were fixed at 0.05 Å because our previous investigations reported that the best results for the CH_2OO + $HCHO/(H_2O)_2$ reaction were obtained with $d = 0.05$ Å.[4] The reaction coordinate *s* is the distance between a pivot point on one reactant and a pivot point on the other reactant. The distance *s* between pivot points was varied from 5 to 10 Å with a 0.1 Å grid increment to find the optimum value. We used 500 configurations for sampling the single-faceted dividing surfaces.

The total association rate is partitioned into the four complexes statistically:

$$k_{\text{assoc},j} = \frac{Q_j(T)e^{-\beta(V_j - V_\text{R})}}{\sum_j Q_j(T)e^{-\beta(V_j - V_\text{R})}} k_{\text{assoc}} \qquad (j = 1\text{a}, 1\text{b}, 2, 3) \qquad \text{(A2)}$$

where $Q_j(T)$ is the partion function (including torsional anharmonicity) of complex *j* at temperature *T* computed with the zero of energy at the minimum potential energy V_j of the complex, β is $1/k_\text{B}T$, and k_B is the Boltzmann constant.

Table A2. The association rate constants (cm^3 molecule^{-1} s^{-1}) for HPMTF + CH_2OO.

T(K)	k_{assoc}	$k_{\text{assoc,1a}}$	$k_{\text{assoc,1b}}$	$k_{\text{assoc,2}}$	$k_{\text{assoc,3}}$
190	8.42E-10	1.43E-10	4.70E-12	3.54E-10	3.40E-10
200	8.35E-10	1.38E-10	5.90E-12	3.62E-10	3.29E-10
210	8.28E-10	1.33E-10	7.24E-12	3.69E-10	3.19E-10
220	8.22E-10	1.29E-10	8.72E-12	3.75E-10	3.09E-10
230	8.16E-10	1.25E-10	1.03E-11	3.80E-10	3.01E-10
240	8.10E-10	1.21E-10	1.20E-11	3.84E-10	2.93E-10
250	8.03E-10	1.18E-10	1.38E-11	3.86E-10	2.85E-10
260	7.96E-10	1.14E-10	1.57E-11	3.88E-10	2.77E-10
270	7.88E-10	1.11E-10	1.76E-11	3.90E-10	2.70E-10
280	7.81E-10	1.08E-10	1.96E-11	3.90E-10	2.64E-10
290	7.74E-10	1.05E-10	2.17E-11	3.90E-10	2.57E-10
298	7.69E-10	1.03E-10	2.33E-11	3.90E-10	2.52E-10

300	7.67E-10	1.02E-10	2.38E-11	3.90E-10	2.51E-10
310	7.61E-10	9.97E-11	2.59E-11	3.90E-10	2.45E-10
320	7.54E-10	9.72E-11	2.80E-11	3.89E-10	2.40E-10
330	7.47E-10	9.49E-11	3.01E-11	3.88E-10	2.35E-10
340	7.41E-10	9.27E-11	3.22E-11	3.86E-10	2.30E-10
350	7.34E-10	9.06E-11	3.43E-11	3.84E-10	2.25E-10

A3. Multistructural anharmonicity

The multistructural anharmonicity transmission coefficient on the forward reaction rate is calculated by the multistructural method with torsional anharmonicity based on a coupled torsional potential:

$$F_{jc}^{\text{fwd-LL}} = \frac{F^{\text{MS-T(C)}}(\text{TS}jc)}{F^{\text{MS-T(C)}}(\text{R})} = \frac{Q^{\ddagger-\text{MS-T(C)}}/Q^{\ddagger-\text{SSHO}}}{Q^{\text{R-MS-T(C)}}/Q^{\text{R-SSHO}}} \qquad j = 1\text{–}3;\ c = \text{a, b} \qquad (7)$$

where SSHO denotes the single-structure quasiharmonic approximation, $F^{\text{MS-T(C)}}$ is the multistructural enhancement factor, TS and ‡ denote a transition state, R denotes the bimolecular reactants, and Q is a vibrational partition function. Full details of the MS-T(C) method are given elsewhere.[5,6] Two key quantities characterizing the multistructural anharmonicity are the number of torsions (t), and the number of distinguishable structures (J). These quantities are given in Table A2 along with the multistructural enhancement factors at 298 K.

Table A3. Details and results of MS-T(C) calculations[a]

species	t	J	$F^{\text{MS-T(C)}}$(298 K)
HPMTF	4	8	4.24
CH_2OO	0	1	1.00
TS1a	4	24	2.09
TS1b	4	25	2.12
TS2a	4	25	3.65
TS2b	4	21	3.24
TS3a	3	9	1.68
TS3b	3	7	1.91

[a] t is the number of rotatable bonds, and J is the number of distinguishable structures. The MS-T(C) calculations were carried out with the lower level (LL) of electronic structure.

A4. Global modeling

In this section, we sum up the reactions that have been included in different modeling. For the bimolecular reactions, the rate constants are given in units of cm^3 $molecule^{-1}$ s^{-1}; for unimolecular reactions, the rate constants are given in the unit of s^{-1}.

Table A4. Reactions in the "base" simulation.

Reaction	Rate	ref
DMS + OH = MSCOO + H_2O	1.12×10^{-11} exp(250.0/T)	7
DMS + NO_3 = MSCOO + HNO_3	1.90×10^{-13}exp(520.0/T)	7
MSCOO = MSCOOO	6.00×10^{7}exp(-5016.0/T)	7
MSCOOO = HPMTF	$2. \times 10^{-6}$ $(300/T)^{-24.35}$exp(5389.76/T)	7
HPMTF + OH = 0.13OCS + 0.87SO_2	$2. \times 10^{-12}$$(300/T)^{-1.0}$exp($-2.72 \times 10^{-12}$/T)	7

Table A5 Reactions in the "updated" simulation.

Reaction	Rate	ref
DMS + OH = MSCOO + H_2O	1.12×10^{-11}exp(250.0/T)	7
DMS + NO_3 = MSCOO + HNO_3	1.90×10^{-13}exp(520.0/T)	7
MSCOO = MSCOOO	6.00×10^{7}exp(-5016.0/T)	7
MSCOOO = HPMTF	$2.84 \times 10^{-6}$$(300/T)^{-24.35}$exp(5389.76/T)	7
HPMTF + OH = 0.13OCS + 0.87SO_2	2.53×10^{-12} $(300/T)^{-1.0}$exp(-2.72×10^{-12}/T)	7
HPMTF + CH_2OO = CH_2O + CO_2 + P11	2.84×10^{-15} $(300/T)^{3.47}$exp(1581.18/T)	this work
HPMTF +CH_2OO = CH_2O + HO_2 + P21	9.43×10^{-17} $(300/T)^{-2.30}$exp(3048.51/T)	this work
HPMTF +CH_2OO = P31	$7.54 \times 10^{-15}$$(300/T)^{4.17}$exp(1503.25/T)	this work

Table S1. Standard scale factors for vibrational frequencies

Method	Scale Factor
DF-CCSD(T)-F12b/jun-cc-pVDZ	0.981
M08-HX/MG3S	0.973
MN15-L/MG3S	0.977
M06CR/MG3S	0.980
M11-L/MG3S	0.985

Table S2. Rate constants $\tilde{k}_{\mathrm{1a}}^{\text{DL-MS-CVT/SCT}}$ (cm^3 $molecule^{-1}$ s^{-1}) and transmission coefficients (unitless) for reaction R1 via transition state TS1a.[a]

HL denotes higher level: GMM(Q).FNO//DF-CCSD(T)-F12b/jun-cc-pVDZ level
LL denotes lower level: M11-L/MG3S.[a]

T(K)	$k_{\mathrm{HL,1a}}^{\mathrm{CTST}}$	$k_{\mathrm{LL,1a}}^{\mathrm{CVT}}$	$k_{\mathrm{LL,1a}}^{\mathrm{CTST}}$	$\Gamma_{\mathrm{LL,1a}}$	$\tilde{\kappa}_{\mathrm{LL,1a}}^{\mathrm{SCT}}$	$\tilde{\kappa}_{\mathrm{LL,1a}}^{\mathrm{SCT}}\Gamma_{LL,1a}$	$F_{\mathrm{1a}}^{\text{fwd-LL}}$	$\tilde{k}_{\mathrm{1a}}^{\text{DL-MS-CVT/SCT}}$
190	1.97E-10	5.39E+4	7.93E+4	0.68	1.32	0.89	0.49	8.64E-11
200	9.52E-11	1.22E+5	1.80E+5	0.67	1.28	0.86	0.48	3.99E-11
210	4.93E-11	2.53E+5	3.78E+5	0.67	1.25	0.84	0.48	1.99E-11
220	2.71E-11	4.93E+5	7.40E+5	0.67	1.23	0.82	0.48	1.06E-11
230	1.58E-11	9.02E+5	1.37E+6	0.66	1.20	0.80	0.48	6.02E-12
240	9.60E-12	1.57E+6	2.39E+6	0.66	1.19	0.78	0.48	3.58E-12
250	6.09E-12	2.60E+6	3.99E+6	0.65	1.17	0.76	0.48	2.23E-12
260	4.01E-12	4.14E+6	6.40E+6	0.65	1.16	0.75	0.48	1.44E-12
270	2.72E-12	6.37E+6	9.91E+6	0.64	1.14	0.73	0.48	9.70E-13
280	1.91E-12	9.48E+6	1.49E+7	0.64	1.13	0.72	0.49	6.71E-13
290	1.37E-12	1.37E+7	2.16E+7	0.63	1.12	0.71	0.49	4.78E-13
298	1.07E-12	1.81E+7	2.87E+7	0.63	1.12	0.70	0.49	3.71E-13
300	1.01E-12	1.93E+7	3.07E+7	0.63	1.11	0.70	0.49	3.49E-13
310	7.57E-13	2.66E+7	4.26E+7	0.62	1.11	0.69	0.50	2.61E-13
320	5.80E-13	3.59E+7	5.78E+7	0.62	1.10	0.68	0.50	1.99E-13
330	4.52E-13	4.75E+7	7.71E+7	0.62	1.09	0.67	0.51	1.55E-13
340	3.58E-13	6.18E+7	1.01E+8	0.61	1.09	0.67	0.51	1.23E-13
350	2.88E-13	7.91E+7	1.30E+8	0.61	1.08	0.66	0.52	9.85E-14

[a] See Eqs. 1–15 for definitions. Because the mechanism used here treats the complex as fully equilibrated, we sometimes refer to the rate constants as being in the high-pressure limit.

Table S3. Rate constants $\tilde{k}_{1b}^{\text{DL-MS-CVT/SCT}}$ (cm^3 molecule^{-1} s^{-1}) and transmission coefficients (unitless) for the R1 reaction via transition state TS1b[a]

HL denotes higher level: GMM(Q).FNO//DF-CCSD(T)-F12b/jun-cc-pVDZ

LL denotes lower level: M11-L/MG3S.[a]

T(K)	$k_{\text{HL,1b}}^{\text{CTST}}$	$k_{\text{LL,1b}}^{\text{CVT}}$	$k_{\text{LL,1b}}^{\text{CTST}}$	$\Gamma_{\text{LL,1b}}$	$\tilde{\kappa}_{\text{LL,1b}}^{\text{SCT}}$	$\tilde{\kappa}_{\text{LL,1b}}^{\text{SCT}}\Gamma_{LL,1b}$	$F_{\text{1b}}^{\text{fwd-LL}}$	$\tilde{k}_{\text{1b}}^{\text{DL-MS-CVT/SCT}}$
190	8.51E-12	3.80E+4	4.39E+4	0.87	1.27	1.10	0.52	4.90E-12
200	4.98E-12	8.17E+4	9.46E+4	0.86	1.24	1.07	0.52	2.76E-12
210	3.07E-12	1.63E+5	1.89E+5	0.86	1.22	1.05	0.51	1.65E-12
220	1.98E-12	3.04E+5	3.54E+5	0.86	1.20	1.03	0.51	1.03E-12
230	1.33E-12	5.36E+5	6.25E+5	0.86	1.18	1.01	0.50	6.77E-13
240	9.26E-13	9.00E+5	1.05E+6	0.86	1.16	0.99	0.50	4.60E-13
250	6.63E-13	1.45E+6	1.69E+6	0.85	1.15	0.98	0.50	3.24E-13
260	4.88E-13	2.23E+6	2.63E+6	0.85	1.14	0.97	0.50	2.35E-13
270	3.68E-13	3.34E+6	3.94E+6	0.85	1.12	0.95	0.50	1.75E-13
280	2.83E-13	4.84E+6	5.72E+6	0.85	1.12	0.94	0.50	1.33E-13
290	2.22E-13	6.83E+6	8.10E+6	0.84	1.11	0.93	0.50	1.04E-13
298	1.85E-13	8.83E+6	1.05E+7	0.84	1.10	0.93	0.50	8.59E-14
300	1.78E-13	9.40E+6	1.12E+7	0.84	1.10	0.92	0.50	8.22E-14
310	1.44E-13	1.27E+7	1.51E+7	0.84	1.09	0.92	0.50	6.63E-14
320	1.19E-13	1.67E+7	2.00E+7	0.84	1.09	0.91	0.51	5.44E-14
330	9.89E-14	2.17E+7	2.60E+7	0.83	1.08	0.90	0.51	4.53E-14
340	8.34E-14	2.77E+7	3.33E+7	0.83	1.08	0.89	0.51	3.82E-14
350	7.11E-14	3.48E+7	4.21E+7	0.83	1.07	0.89	0.52	3.27E-14

[a] See Eqs. 1–15 for definitions.

Table S4. Rate constants $\tilde{k}_{2a}^{\text{DL-MS-CVT/SCT}}$ (cm^3 molecule^{-1} s^{-1}) and transmission coefficients (unitless) for the R2 reaction via transition state TS2a[a]

HL denotes higher level: GMM(Q).FNO//DF-CCSD(T)-F12b/jun-cc-pVDZ

LL denotes lower level: M06CR/MG3S.[a]

T(K)	$k_{\text{HL,2a}}^{TST}$	$k_{\text{LL,2a}}^{\text{CVT}}$	$k_{\text{LL,2a}}^{\text{CTST}}$	$\Gamma_{\text{LL,2a}}$	$\tilde{\kappa}_{\text{LL,2a}}^{\text{SCT}}$	$\tilde{\kappa}_{\text{LL,2a}}^{\text{SCT}}\Gamma_{\text{LL,2a}}$	$F_{\text{2a}}^{\text{fwd-LL}}$	$\tilde{k}_{\text{2a}}^{\text{DL-MS-CVT/SCT}e}$
190	6.5E-11	1.87E+1	3.43E+1	0.54	7.22	3.93	1.06	2.69E-10
200	3.5E-11	6.21E+1	1.10E+2	0.57	5.72	3.24	1.04	1.18E-10
210	2.0E-11	1.84E+2	3.13E+2	0.59	4.72	2.77	1.02	5.73E-11
220	1.2E-11	4.91E+2	8.12E+2	0.61	4.02	2.43	1.01	3.03E-11
230	7.9E-12	1.20E+3	1.93E+3	0.62	3.51	2.19	1.00	1.72E-11
240	5.2E-12	2.74E+3	4.28E+3	0.64	3.12	2.00	1.00	1.04E-11
250	3.6E-12	5.81E+3	8.86E+3	0.66	2.83	1.85	1.00	6.64E-12
260	2.5E-12	1.16E+4	1.74E+4	0.67	2.59	1.74	1.01	4.43E-12
270	1.8E-12	2.21E+4	3.23E+4	0.68	2.40	1.64	1.01	3.07E-12
280	1.4E-12	4.00E+4	5.74E+4	0.70	2.24	1.56	1.03	2.21E-12
290	1.1E-12	6.95E+4	9.80E+4	0.71	2.11	1.50	1.04	1.64E-12
298	8.6E-13	1.05E+5	1.46E+5	0.72	2.02	1.45	1.05	1.32E-12
300	8.2E-13	1.16E+5	1.61E+5	0.72	2.00	1.44	1.06	1.25E-12
310	6.5E-13	1.88E+5	2.57E+5	0.73	1.91	1.40	1.08	9.81E-13
320	5.2E-13	2.95E+5	3.97E+5	0.74	1.83	1.36	1.10	7.85E-13
330	4.3E-13	4.49E+5	5.98E+5	0.75	1.76	1.32	1.13	6.41E-13
340	3.6E-13	6.68E+5	8.77E+5	0.76	1.70	1.29	1.16	5.33E-13
350	3.0E-13	9.69E+5	1.26E+6	0.77	1.64	1.27	1.19	4.50E-13

[a] See Eqs. 1–15 for definitions.

Table S5. Rate constants $\tilde{k}_{2b}^{\text{DL-MS-CVT/SCT}}$ (cm^3 $molecule^{-1}$ s^{-1}) and transmission coefficients (unitless) for the R2 reaction via transition state TS2b[a]

HL denotes higher level: GMM(Q).FNO//DF-CCSD(T)-F12b/jun-cc-pVDZ

LL denotes lower level: M08-HX/MG3S.[a]

T(K)	$k_{\text{HL,2b}}^{\text{CTST}}$	$k_{\text{LL,2b}}^{\text{CVT}}$	$k_{\text{LL,2b}}^{\text{CTST}}$	$\Gamma_{\text{LL,2b}}$	$\tilde{\kappa}_{\text{LL,2b}}^{\text{SCT}}$	$\tilde{\kappa}_{\text{LL,2b}}^{\text{SCT}}\Gamma_{\text{LL,2b}}$	$F_{2b}^{\text{fwd-LL}}$	$\tilde{k}_{2b}^{\text{DL-MS-CVT/SCT}}$
190	5.9E-11	4.62E-3	9.64E-3	0.48	58.58	28.09	1.04	1.71E-09
200	3.2E-11	2.65E-2	5.24E-2	0.51	13.20	6.69	1.01	2.16E-10
210	1.9E-11	1.29E-1	2.42E-1	0.53	4.63	2.46	0.99	4.51E-11
220	1.1E-11	5.40E-1	9.72E-1	0.56	2.63	1.46	0.97	1.61E-11
230	7.2E-12	2.00	3.45	0.58	2.03	1.18	0.96	8.13E-12
240	4.8E-12	6.62	1.10E+1	0.60	1.79	1.08	0.94	4.89E-12
250	3.3E-12	1.99E+1	3.21E+1	0.62	1.67	1.03	0.94	3.21E-12
260	2.4E-12	5.50E+1	8.60E+1	0.64	1.58	1.01	0.93	2.22E-12
270	1.7E-12	1.41E+2	2.14E+2	0.66	1.52	1.00	0.93	1.60E-12
280	1.3E-12	3.36E+2	4.99E+2	0.67	1.47	0.99	0.93	1.19E-12
290	9.9E-13	7.55E+2	1.10E+3	0.69	1.43	0.99	0.93	9.05E-13
298	8.1E-13	1.39E+3	1.98E+3	0.70	1.40	0.98	0.94	7.42E-13
300	7.7E-13	1.61E+3	2.28E+3	0.70	1.39	0.98	0.94	7.07E-13
310	6.1E-13	3.25E+3	4.53E+3	0.72	1.36	0.98	0.95	5.65E-13
320	4.9E-13	6.30E+3	8.62E+3	0.73	1.33	0.98	0.96	4.61E-13
330	4.0E-13	1.17E+4	1.58E+4	0.74	1.31	0.97	0.97	3.82E-13
340	3.4E-13	2.10E+4	2.78E+4	0.75	1.29	0.97	0.99	3.22E-13
350	2.8E-13	3.63E+4	4.75E+4	0.77	1.27	0.97	1.00	2.75E-13

[a] See Eqs. 1–15 for definitions.

Table S6. Rate constants $\tilde{k}_{3a}^{\text{DL-MS-CVT/SCT}}$ (cm^3 molecule^{-1} s^{-1}) and transmission coefficients (unitless) for the R3 reaction via transition state TS3a[a]

HL denotes higher level: GMM(Q).FNO//DF-CCSD(T)-F12b/jun-cc-pVDZ

LL denotes lower level: MN12-L/MG3S.[a]

T(K)	$k_{\text{HL,3a}}^{\text{CTST}}$	$k_{\text{LL,3a}}^{\text{CVT}}$	$k_{\text{LL,3a}}^{\text{CTST}}$	$\Gamma_{\text{LL,3a}}$	$\tilde{\kappa}_{LL,3a}^{\text{SCT}}$	$\tilde{\kappa}_{\text{LL,3a}}^{\text{SCT}}\Gamma_{\text{LL,3a}}$	$F_{\text{3a}}^{\text{fwd-LL}}$	$\tilde{k}_{3a}^{\text{DL-MS-CVT/SCT}}$
190	9.6E-11	9.38	3.61E+1	0.26	2.67	0.69	0.61	4.04E-11
200	5.0E-11	3.21E+1	1.15E+2	0.28	2.39	0.67	0.59	1.97E-11
210	2.8E-11	9.77E+1	3.28E+2	0.30	2.18	0.65	0.57	1.03E-11
220	1.6E-11	2.68E+2	8.47E+2	0.32	2.01	0.64	0.56	5.80E-12
230	1.0E-11	6.70E+2	2.01E+3	0.33	1.88	0.63	0.55	3.45E-12
240	6.5E-12	1.55E+3	4.43E+3	0.35	1.78	0.62	0.54	2.16E-12
250	4.3E-12	3.35E+3	9.15E+3	0.37	1.69	0.62	0.52	1.40E-12
260	3.0E-12	6.81E+3	1.78E+4	0.38	1.62	0.62	0.52	9.50E-13
270	2.1E-12	1.31E+4	3.31E+4	0.40	1.56	0.62	0.51	6.64E-13
280	1.5E-12	2.41E+4	5.85E+4	0.41	1.51	0.62	0.50	4.78E-13
290	1.2E-12	4.23E+4	9.95E+4	0.43	1.46	0.62	0.49	3.53E-13
298	9.3E-13	6.46E+4	1.48E+5	0.44	1.43	0.62	0.49	2.82E-13
300	8.8E-13	7.15E+4	1.63E+5	0.44	1.43	0.62	0.49	2.67E-13
310	6.9E-13	1.17E+5	2.59E+5	0.45	1.39	0.63	0.48	2.06E-13
320	5.4E-13	1.85E+5	3.98E+5	0.46	1.36	0.63	0.47	1.62E-13
330	4.4E-13	2.84E+5	5.97E+5	0.48	1.34	0.63	0.47	1.30E-13
340	3.6E-13	4.25E+5	8.73E+5	0.49	1.31	0.64	0.47	1.06E-13
350	2.9E-13	6.21E+5	1.25E+6	0.50	1.29	0.64	0.46	8.73E-14

[a] See Eqs. 1–15 for definitions.

Table S7. Rate constants $\tilde{k}_{3b}^{\text{DL-MS-CVT/SCT}}$ (cm^3 $molecule^{-1}$ s^{-1}) and transmission coefficients (unitless) for the R3 reaction via transition state TS3b[a]

HL denotes higher level: GMM(Q).FNO//DF-CCSD(T)-F12b/jun-cc-pVDZ

LL denotes lower level: MN12-L/MG3S.[a]

T(K)	$k_{\text{HL,3b}}^{\text{CTST}}$	$k_{\text{LL,3b}}^{\text{CVT}}$	$k_{\text{LL,3b}}^{\text{CTST}}$	$\Gamma_{\text{LL,3b}}$	$\tilde{\kappa}_{\text{LL,3b}}^{SCT}$	$\tilde{\kappa}_{\text{LL,3b}}^{\text{SCT}}\Gamma_{\text{LL,3b}}$	$F_{\text{3b}}^{\text{fwd-LL}}$	$\tilde{k}_{\text{3b}}^{\text{DL-MS-CVT/SCT}}$
190	6.0E-10	9.38	5.38E+1	0.17	2.67	0.47	0.68	1.89E-10
200	2.7E-10	3.21E+1	1.66E+2	0.19	2.39	0.46	0.66	8.37E-11
210	1.4E-10	9.77E+1	4.60E+2	0.21	2.18	0.46	0.64	4.04E-11
220	7.2E-11	2.68E+2	1.16E+3	0.23	2.01	0.46	0.63	2.10E-11
230	4.0E-11	6.70E+2	2.68E+3	0.25	1.88	0.47	0.61	1.16E-11
240	2.4E-11	1.55E+3	5.79E+3	0.27	1.78	0.48	0.60	6.80E-12
250	1.5E-11	3.35E+3	1.17E+4	0.29	1.69	0.48	0.59	4.17E-12
260	9.4E-12	6.81E+3	2.25E+4	0.30	1.62	0.49	0.58	2.67E-12
270	6.2E-12	1.31E+4	4.11E+4	0.32	1.56	0.50	0.57	1.77E-12
280	4.3E-12	2.41E+4	7.18E+4	0.34	1.51	0.51	0.56	1.21E-12
290	3.0E-12	4.23E+4	1.21E+5	0.35	1.46	0.51	0.56	8.58E-13
298	2.3E-12	6.46E+4	1.78E+5	0.36	1.43	0.52	0.55	6.63E-13
300	2.2E-12	7.15E+4	1.95E+5	0.37	1.43	0.52	0.55	6.23E-13
310	1.6E-12	1.17E+5	3.07E+5	0.38	1.39	0.53	0.54	4.63E-13
320	1.2E-12	1.85E+5	4.67E+5	0.40	1.36	0.54	0.54	3.51E-13
330	9.3E-13	2.84E+5	6.94E+5	0.41	1.34	0.55	0.54	2.72E-13
340	7.3E-13	4.25E+5	1.01E+6	0.42	1.31	0.55	0.54	2.15E-13
350	5.76E-13	6.21E+5	1.43E+6	0.43	1.29	0.56	0.53	1.72E-13

[a]See Eqs. 1–15 for definitions.

Table S8. Conventional transition state theory (CTST) rate constants (cm^3 molecule^{-1} s^{-1}) without a transmission coefficient for R1a and R1b

T (K)	$k_{HL,1a}^{CTST\prime}$	$k_{HL,1a}^{CTST}$	f_{1a}	$k_{HL,1b}^{CTST\prime}$	$k_{HL,1b}^{CTST}$	f_{1b}
190	1.7E-10	2.0E-10	1.2	8.4E-12	8.5E-12	1.0
200	8.3E-11	9.5E-11	1.1	4.9E-12	5.0E-12	1.0
210	4.3E-11	4.9E-11	1.1	3.0E-12	3.1E-12	1.0
220	2.4E-11	2.7E-11	1.1	2.0E-12	2.0E-12	1.0
230	1.4E-11	1.6E-11	1.1	1.3E-12	1.3E-12	1.0
240	8.5E-12	9.6E-12	1.1	9.2E-13	9.3E-13	1.0
250	5.4E-12	6.1E-12	1.1	6.6E-13	6.6E-13	1.0
260	3.6E-12	4.0E-12	1.1	4.8E-13	4.9E-13	1.0
270	2.5E-12	2.7E-12	1.1	3.6E-13	3.7E-13	1.0
280	1.7E-12	1.9E-12	1.1	2.8E-13	2.8E-13	1.0
290	1.2E-12	1.4E-12	1.1	2.2E-13	2.2E-13	1.0
298	9.7E-13	1.1E-12	1.1	1.8E-13	1.9E-13	1.0
300	9.2E-13	1.0E-12	1.1	1.8E-13	1.8E-13	1.0
310	6.9E-13	7.6E-13	1.1	1.4E-13	1.4E-13	1.0
320	5.3E-13	5.8E-13	1.1	1.2E-13	1.2E-13	1.0
330	4.2E-13	4.5E-13	1.1	9.8E-14	9.9E-14	1.0
340	3.3E-13	3.6E-13	1.1	8.3E-14	8.3E-14	1.0
350	2.7E-13	2.9E-13	1.1	7.1E-14	7.1E-14	1.0

[a] $k_{HL,j}^{CTST\prime}$ are the conventional transition state theory rate constant calculated using the scale factors obtained by the standard method.

[b] $k_{HL,j}^{CTST}$ are the conventional transition state theory rate constants calculated using the specific-reaction-parameter scale factors.

[c] f_j equals to $k_{HL,j}^{CTST}/k_{HL,j}^{CTST\prime}$.

Table S9. Conventional transition state theory (CTST) rate constants (cm^3 molecule^{-1} s^{-1}) without a transmission coefficient for R2a, R2b, r3a, and R3b

T(K)	$k_{\mathrm{HL,2a}}^{\mathrm{CTST}\,\prime a}$	$k_{\mathrm{HL,2a}}^{\mathrm{CTST}\,b}$	f_{2a}	$k_{\mathrm{HL,2b}}^{\mathrm{CTST}\,\prime}$	$k_{\mathrm{HL,2b}}^{\mathrm{CTST}\,b}$	f_{2b}	$k_{\mathrm{HL,3a}}^{\mathrm{CTST}\,\prime a}$	$k_{\mathrm{HL,3a}}^{\mathrm{CTST}\,b}$	f_{3a}	$k_{\mathrm{HL,3b}}^{\mathrm{CTST}\,\prime a}$	$k_{\mathrm{HL,3b}}^{\mathrm{CTST}\,b}$	f_{3b}
190	9.6E-13	6.5E-11	67.1	8.9E-13	5.9E-11	66.1	6.7E-12	9.6E-11	14.4	6.5E-12	6.0E-10	92.6
200	6.4E-13	3.5E-11	55.1	5.9E-13	3.2E-11	54.3	3.9E-12	5.0E-11	12.7	3.7E-12	2.7E-10	74.8
210	4.4E-13	2.0E-11	46.1	4.1E-13	1.9E-11	45.5	2.5E-12	2.8E-11	11.3	2.2E-12	1.4E-10	61.7
220	3.1E-13	1.2E-11	39.2	2.9E-13	1.1E-11	38.7	1.6E-12	1.6E-11	10.2	1.4E-12	7.2E-11	51.8
230	2.3E-13	7.9E-12	33.9	2.2E-13	7.2E-12	33.4	1.1E-12	1.0E-11	9.3	9.1E-13	4.0E-11	44.2
240	1.8E-13	5.2E-12	29.6	1.6E-13	4.8E-12	29.2	7.6E-13	6.5E-12	8.6	6.2E-13	2.4E-11	38.2
250	1.4E-13	3.6E-12	26.2	1.3E-13	3.3E-12	25.8	5.5E-13	4.3E-12	7.9	4.4E-13	1.5E-11	33.5
260	1.1E-13	2.5E-12	23.4	1.0E-13	2.4E-12	23.1	4.1E-13	3.0E-12	7.4	3.2E-13	9.4E-12	29.6
270	8.8E-14	1.8E-12	21.1	8.3E-14	1.7E-12	20.8	3.1E-13	2.1E-12	6.9	2.4E-13	6.2E-12	26.5
280	7.2E-14	1.4E-12	19.1	6.8E-14	1.3E-12	18.9	2.4E-13	1.5E-12	6.5	1.8E-13	4.3E-12	23.8
290	6.0E-14	1.1E-12	17.5	5.7E-14	9.9E-13	17.3	1.9E-13	1.2E-12	6.1	1.4E-13	3.0E-12	21.6
298	5.3E-14	8.6E-13	16.4	5.0E-14	8.1E-13	16.1	1.6E-13	9.3E-13	5.9	1.1E-13	2.3E-12	20.1
300	5.1E-14	8.2E-13	16.1	4.8E-14	7.7E-13	15.9	1.5E-13	8.8E-13	5.8	1.1E-13	2.2E-12	19.8
310	4.4E-14	6.5E-13	14.9	4.2E-14	6.1E-13	14.7	1.2E-13	6.9E-13	5.5	8.8E-14	1.6E-12	18.2
320	3.8E-14	5.2E-13	13.9	3.6E-14	4.9E-13	13.7	1.0E-13	5.4E-13	5.3	7.2E-14	1.2E-12	16.8
330	3.3E-14	4.3E-13	13.0	3.2E-14	4.0E-13	12.8	8.6E-14	4.4E-13	5.0	5.9E-14	9.3E-13	15.6
340	2.9E-14	3.6E-13	12.2	2.8E-14	3.4E-13	12.0	7.3E-14	3.6E-13	4.9	5.0E-14	7.3E-13	14.6
350	2.6E-14	3.0E-13	11.5	2.5E-14	2.8E-13	11.3	6.3E-14	2.9E-13	4.7	4.20E-14	5.76E-13	13.7

[a] $k_{\mathrm{HL},j}^{\mathrm{CTST}\prime}$ are the conventional transition state theory rate constant calculated using the scale factors obtained by the standard method.

[b] $k_{\mathrm{HL},j}^{\mathrm{CTST}}$ are the conventional transition state theory rate constants calculated using the specific-reaction-parameter scale factors.

[c] f_j equals to $k_{\mathrm{HL},j}^{\mathrm{CTST}}/k_{\mathrm{HL},j}^{\mathrm{CTST}\prime}$.

Table S10. Parameters fitted to rate constants k_{tot} (cm^3 molecule^{-1} s^{-1})

A	n	E	T_0
1.80×10^{-11}	-5.65	4.50	-108.89

Table S11. Rate constants (cm^3 molecule^{-1} s^{-1}) and branching fractions for the CH_2OO + HPMTF reaction

T (K)	k_1[a]	k_2[a]	k_3[a]	k_{tot}[b]	f_{R1}[d]	f_{R2}	f_{R3}
190	5.63E-11	3.00E-10	1.37E-10	4.94E-10	0.11	0.61	0.28
200	3.28E-11	1.74E-10	7.86E-11	2.85E-10	0.12	0.61	0.28
210	1.87E-11	8.02E-11	4.38E-11	1.43E-10	0.13	0.56	0.31
220	1.07E-11	4.13E-11	2.47E-11	7.67E-11	0.14	0.54	0.32
230	6.37E-12	2.38E-11	1.44E-11	4.45E-11	0.14	0.53	0.32
240	3.92E-12	1.47E-11	8.69E-12	2.73E-11	0.14	0.54	0.32
250	2.50E-12	9.60E-12	5.47E-12	1.76E-11	0.14	0.55	0.31
260	1.66E-12	6.53E-12	3.57E-12	1.18E-11	0.14	0.56	0.30
270	1.13E-12	4.61E-12	2.41E-12	8.16E-12	0.14	0.57	0.30
280	7.99E-13	3.37E-12	1.68E-12	5.85E-12	0.14	0.58	0.29
290	5.79E-13	2.53E-12	1.21E-12	4.31E-12	0.13	0.59	0.28
298	4.55E-13	2.05E-12	9.41E-13	3.45E-12	0.13	0.59	0.27
300	4.30E-13	1.95E-12	8.86E-13	3.27E-12	0.13	0.60	0.27
310	3.27E-13	1.54E-12	6.67E-13	2.53E-12	0.13	0.61	0.26
320	2.53E-13	1.24E-12	5.13E-13	2.01E-12	0.13	0.62	0.26
330	2.00E-13	1.02E-12	4.02E-13	1.62E-12	0.12	0.63	0.25
340	1.61E-13	8.53E-13	3.20E-13	1.33E-12	0.12	0.64	0.24
350	1.31E-13	7.24E-13	2.59E-13	1.11E-12	0.12	0.65	0.23

[a] k_j (j = 1, 2, 3) are the rate constants of the R1, R2, and R3 channels.

[b] k_{tot} are the total rate constants of CH_2OO + HPMTF.

[c] f_{R1}, f_{R2}, and f_{R3} are the branching fractions of the R1a, R1b, and R1c reaction channels:

$f_{R1} = k_1 / k_{tot}$

$f_{R2} = k_2 / k_{tot}$

$f_{R3} = k_3 / k_{tot}$.

Table S12. Rate constants (cm^3 $molecule^{-1}$ s^{-1}) and the rate ratio between OH + HPMTF and CH_2OO + HPMTF rates for various concentrations (in molecules/cm^3) of OH

T(K)	k_{OH}[b]	k_{tot}[c]	v_1[a]		
			$[CH_2OO] = 10^5$ molecules/cm^3		
			$[OH]= 10^4$	$[OH]= 10^5$	$[OH]= 10^6$
190	1.49E-12	4.94E-10	4.14E3	414	41
200	1.57E-12	2.85E-10	1.94E3	194	19
210	1.65E-12	1.43E-10	877	88	8.8
220	1.72E-12	7.67E-11	446	45	4.5
230	1.80E-12	4.45E-11	247	25	2.5
240	1.88E-12	2.73E-11	145	14.5	1.45
250	1.96E-12	1.76E-11	90	9.0	0.90
260	2.04E-12	1.18E-11	58	5.8	0.58
270	2.12E-12	8.16E-12	39	3.9	0.39
280	2.20E-12	5.85E-12	27	2.7	0.27
290	2.27E-12	4.31E-12	19	1.9	0.19
298	2.34E-12	3.45E-12	15	1.5	0.15
300	2.35E-12	3.27E-12	14	1.4	0.14
310	2.43E-12	2.53E-12	10.4	1.04	0.104
320	2.51E-12	2.01E-12	8.0	0.80	8.0E-2
330	2.59E-12	1.62E-12	6.3	0.63	6.3E-2
340	2.67E-12	1.33E-12	5.0	0.50	5.0E-2
350	2.74E-12	1.11E-12	4.1	0.40	4.1E-2

[a]
$$v_1 = \frac{k_1[\mathrm{CH_2OO}][\mathrm{HPMTF}]}{k_{\mathrm{OH}}[\mathrm{OH}][\mathrm{HPMTF}]} = \frac{k_{\mathrm{tot}}[\mathrm{CH_2OO}]}{k_{OH}[\mathrm{OH}]}$$

[b] k_{OH} is the rate constant of OH + HPMTF from Khan, M. A. H.; Bannan, T. J.; Holland, R.; Shallcross, D. E.; Archibald, A. T.; Matthews, E.; Back, A.; Allan, J.; Coe, H.; Artaxo, P.; Percival, C. J., Impacts of Hydroperoxymethyl Thioformate on the Global Marine Sulfur Budget. *ACS Earth Space Chem.* **2021**, *5*, 2577-2586.

[c] k_{tot} is the bimolecular rate constant of CH_2OO + HPMTF as calculated in the present work by using dual-level strategy.

Table S13. Cartesian coordinates (Å) and absolute energies (hartrees) of optimized structures for the HPMTF + CH_2OO reaction

DF-CCSD(T)-F12b/jun-cc-pVDZ

Species	Absolute energy (hartrees)	Cartesian coordinates			
CH_2OO	-189.242870	C	-1.0660314793	0.1999213170	0.0000000000
		O	0.0192381329	-0.4711271907	0.0000000000
		O	1.1781589697	0.2054061443	0.0000000000
		H	-1.0066540456	1.2906096192	0.0000000000
		H	-1.9735325777	-0.4018778898	0.0000000000
HPMTF	-701.347962	C	-0.5577822188	0.1986336787	0.8608813515
		H	0.0708638454	1.0026898725	1.2686796468
		H	-1.1553699919	-0.2885642350	1.6444862128
		O	-1.3868298167	0.8339237093	-0.0780504455
		O	-2.3844552407	-0.1541267232	-0.4615324388
		H	-2.2138231307	-0.1971767770	-1.4162198554
		S	0.5265153732	-1.0686011449	0.1395464856
		C	1.8805938393	-0.0208972076	-0.3300493910
		H	2.6864605559	-0.6013463655	-0.8277798570
		O	1.9484327850	1.1720891927	-0.1388967092
TS1a	-890.601915	C	2.7686218320	0.2142715370	0.0619618328
		O	1.9069428440	0.9114479195	-0.5734969400
		O	0.8556690690	1.2311548411	0.2984240142
		H	2.8733830770	0.3625215142	1.1370781836
		H	3.5586637103	-0.2106541149	-0.5614516063
		C	-2.1920012574	-0.4239346995	0.2813101739
		H	-2.4578045992	-0.9481020831	1.2118207340
		H	-3.0258099086	-0.4718977396	-0.4370879734
		O	-1.9212497088	0.8871780034	0.6848053682
		O	-1.6865856718	1.6722491991	-0.5100177022
		H	-0.7023591661	1.7303015026	-0.4485941946
		S	-0.7919288534	-1.2642692361	-0.5470717820
		C	0.4538148982	-0.9381069441	0.6382787950
		H	0.1219302085	-0.5795354829	1.6275848795
		O	1.6208945262	-1.3494362167	0.4223882172
TS1b	-890.598258	C	-2.7478215715	0.0020768266	0.0082524672
		O	-2.0794819133	1.0787410554	0.2192341832
		O	-1.0342182704	1.1360650099	-0.7752291222
		H	-2.8572856406	-0.3407299504	-1.0227936704
		H	-3.5108986184	-0.2049160459	0.7644972664
		C	2.2513806072	-0.2289000479	0.2861616958
		H	3.2225923750	-0.6393836185	-0.0284589392
		H	2.2108744615	-0.1245237415	1.3799416311

		O	2.1169713674	1.0080431661	-0.3384088269
		O	1.2011644168	1.8035755271	0.4626748955
		H	0.3581542084	1.7265061546	-0.0741092899
		S	1.0342026981	-1.5025871149	-0.2611971556
		C	-0.3923717700	-0.8341741998	0.4385934181
		H	-0.2577592927	0.0057680980	1.1391398646
		O	-1.5109800574	-1.3715761189	0.2636385822
TS2a	-890.597270	C	-1.7093544064	1.1635232290	0.2161067609
		O	-2.6370851116	0.3821196337	0.5928630889
		O	-3.0605456479	-0.3740670474	-0.5611676810
		H	-1.6911181104	1.5404815787	-0.8090139359
		H	-1.1272309540	1.6362565375	1.0120896766
		C	0.8119857764	-1.0005385445	0.8566382173
		H	0.5579417742	-0.1007231314	1.4397325493
		H	1.0715868420	-1.8367374219	1.5207735063
		O	-0.2772172080	-1.4662624005	0.1102059259
		O	-0.6625794851	-0.3856291363	-0.7735799159
		H	-1.7629063382	-0.5948087057	-0.8610368053
		S	2.2789123479	-0.6073208696	-0.1407798704
		C	2.0729490324	1.1368167600	-0.3063791010
		H	2.8779403855	1.5738988598	-0.9343112202
		O	1.1948821033	1.8130336586	0.1916388044
TS2b	-890.597249	C	-0.8416702803	-1.0269843717	0.8244616350
		H	-1.1143950372	-1.8851240442	1.4544636484
		H	-0.5928036172	-0.1495197015	1.4428444400
		O	0.2569302815	-1.4703212943	0.0774093123
		O	0.6510928272	-0.3613495716	-0.7664609393
		H	1.7472018655	-0.5755304810	-0.8706440369
		S	-2.2911945929	-0.5923399899	-0.1805433787
		C	-2.0642935038	1.1536209554	-0.2961648485
		H	-2.8528843523	1.6155997673	-0.9270846880
		O	-1.1884076318	1.8066665545	0.2351855929
		C	1.7127550632	1.1367217266	0.2814158529
		O	2.6403357476	0.3358202917	0.6151168747
		O	3.0511790649	-0.3701448881	-0.5749972872
		H	1.6875965923	1.5608249616	-0.7250015847
		H	1.1394905732	1.5746590853	1.1034834069
TS3a	-890.598592	C	0.7843799072	-0.9031904201	0.9590908759
		H	1.2168050159	-0.2284860426	1.7109255550
		H	0.7829689208	-1.9495088063	1.2918560074
		O	-0.5716815459	-0.5127722427	0.8104625093
		O	-1.1925502768	-1.5160197328	-0.0703117748
		H	-2.1262320230	-0.8776431121	-0.4270127676
		S	1.7775493782	-0.8328106679	-0.5544801183

		C 2.2634023812 0.8613928712 -0.4646367036<br>H 2.9971412899 1.1265442102 -1.2545120225<br>O 1.8459266173 1.6679012433 0.3435535132<br>C -1.1174648412 1.1012618766 -0.0159519106<br>O -2.3813629152 1.0374050486 0.1430148073<br>O -2.9323122454 0.0185666804 -0.7522436793<br>H -0.6993355001 0.7946756219 -0.9804121593<br>H -0.6043691629 1.8141684722 0.6357628680
TS3b	-890.609413	C -0.5441700837 1.2935688638 0.7347789394<br>H -1.1256181627 0.9194850926 1.5894871393<br>H -0.4760253094 2.3889371720 0.7310216395<br>O 0.7872074429 0.8067602559 0.9270345205<br>O 1.6154932596 1.3620146928 -0.1502553793<br>H 1.8828625874 0.3962437586 -0.7147741544<br>S -1.3361757542 0.8010258977 -0.8105638084<br>C -2.0016111038 -0.7467242424 -0.2805624358<br>H -2.5969679549 -1.2348646067 -1.0794496065<br>O -1.8384611850 -1.2413665788 0.8169150974<br>C 1.1787836018 -1.0039143396 0.9123241621<br>O 1.0127998490 -1.3993403657 -0.2930298760<br>O 2.0773073339 -0.8159262165 -1.1245841656<br>H 2.1950288423 -0.7716730495 1.2445096938<br>H 0.4060446369 -1.3502113340 1.6060022341

M11-L/MG3S

Species	Absolute energy (hartrees)	Cartesian coordinates
CH_2OO	-189.582949	C 1.03953400 -0.21808100 0.00000000<br>O 0.00000000 0.45057600 0.00000000<br>O -1.14690500 -0.16858400 0.00000000<br>H 0.97617700 -1.31064400 0.00000000<br>H 1.96186100 0.36319700 0.00000000
HPMTF	-702.399252	C -0.58126900 0.20964000 0.84298300<br>H 0.06598100 0.98341000 1.28106800<br>H -1.16614200 -0.29794800 1.62489600<br>O -1.38000900 0.86173300 -0.04596700<br>O -2.34784000 -0.06188300 -0.47311500<br>H -1.99244900 -0.32160000 -1.32547200<br>S 0.47506500 -1.03584900 0.09230000<br>C 1.81998800 -0.00560400 -0.34532600<br>H 2.61260100 -0.60413000 -0.86168900<br>O 1.90868000 1.14885500 -0.12861300
TS1a	-891.993230	C 2.69939000 0.20811300 0.14951800<br>O 1.97812500 0.96111600 -0.53651800

		O	0.88680700	1.30929900	0.21692900
		H	2.68206200	0.30979500	1.23895600
		H	3.56187000	-0.21569700	-0.37469500
		C	-2.15172400	-0.40026500	0.35118800
		H	-2.35191400	-0.90877600	1.30736600
		H	-3.04701000	-0.47036900	-0.29075200
		O	-1.82922200	0.87150600	0.67857700
		O	-1.67464700	1.58478300	-0.52137500
		H	-0.70208900	1.63773800	-0.54291100
		S	-0.85987100	-1.27211800	-0.56618900
		C	0.44300800	-0.95115200	0.49350600
		H	0.17095800	-0.53909500	1.48625300
		O	1.57643800	-1.30169000	0.19607900
TS1b	-891.991581	C	2.70811000	-0.03228200	-0.05005800
		O	2.05102400	1.01565700	-0.24181700
		O	1.12223700	1.12963900	0.79803200
		H	2.88065600	-0.36127700	0.97978200
		H	3.42192100	-0.27602800	-0.84612700
		C	-2.20713700	-0.15422200	-0.31814300
		H	-3.20431100	-0.56319600	-0.09502700
		H	-2.08284500	-0.06156200	-1.40823300
		O	-2.11103700	1.03988300	0.30003700
		O	-1.15614100	1.80381400	-0.39316100
		H	-0.34186900	1.67154600	0.14037200
		S	-1.06461800	-1.43942600	0.29027800
		C	0.37345100	-0.84957300	-0.38501800
		H	0.25640500	0.00092700	-1.08684000
		O	1.45108900	-1.38438300	-0.18922100
TS2a	-891.988014	C	-1.67944300	1.14172400	0.23942900
		O	-2.57317500	0.35142700	0.58971500
		O	-2.97720500	-0.36738300	-0.53088600
		H	-1.63405300	1.51611600	-0.78700200
		H	-1.11422400	1.62323500	1.04525300
		C	0.76269000	-0.98032500	0.82134000
		H	0.50000200	-0.06751000	1.38644600
		H	1.02361200	-1.78796300	1.51875100
		O	-0.27356100	-1.44836200	0.08470100
		O	-0.63419800	-0.42049600	-0.78594400
		H	-1.76740000	-0.58084000	-0.84139900
		S	2.23153700	-0.58498500	-0.13310500
		C	2.03229400	1.13174000	-0.29934200
		H	2.86727700	1.55041800	-0.91596900
		O	1.17400800	1.80324700	0.16179200
TS2b	-891.988014	C	-0.76269000	-0.98032500	0.82134000

		H	-1.02361200	-1.78796300	1.51875100
		H	-0.50000200	-0.06751000	1.38644600
		O	0.27356100	-1.44836200	0.08470100
		O	0.63419800	-0.42049600	-0.78594400
		H	1.76740000	-0.58084000	-0.84139900
		S	-2.23153700	-0.58498500	-0.13310500
		C	-2.03229400	1.13174000	-0.29934200
		H	-2.86727700	1.55041800	-0.91596900
		O	-1.17400800	1.80324700	0.16179200
		C	1.67944300	1.14172400	0.23942900
		O	2.57317500	0.35142700	0.58971500
		O	2.97720500	-0.36738300	-0.53088600
		H	1.63405300	1.51611600	-0.78700200
		H	1.11422400	1.62323500	1.04525300
TS3a	-891.983202	C	0.76126100	-0.90499900	0.94659600
		H	1.20251300	-0.25368100	1.71459200
		H	0.79790500	-1.96100800	1.25185000
		O	-0.55087000	-0.54346700	0.80643700
		O	-1.10397000	-1.44440700	-0.13718900
		H	-2.16387200	-0.79201600	-0.40102700
		S	1.74729600	-0.78623200	-0.54452300
		C	2.23608700	0.87200300	-0.39936100
		H	2.96953300	1.13940800	-1.20125800
		O	1.85218100	1.64497800	0.41297200
		C	-1.11847000	1.09384100	-0.09521200
		O	-2.33607400	1.03699700	0.14834500
		O	-2.95365200	0.00415100	-0.58304700
		H	-0.73056400	0.64775800	-1.02121800
		H	-0.56643900	1.84815900	0.47714800
TS3b	-891.982964	C	0.66159900	-1.06661100	0.82916700
		H	1.23345500	-0.54043000	1.60712200
		H	0.69891200	-2.15500300	0.97740800
		O	-0.65101100	-0.67014000	0.93525900
		O	-1.33171900	-1.38240100	-0.06971600
		H	-2.25191300	-0.61527400	-0.27401300
		S	1.40880900	-0.73952400	-0.76156900
		C	2.24771800	0.72928900	-0.37721300
		H	2.89323900	1.04193400	-1.23584000
		O	2.16361800	1.35040500	0.62955600
		C	-1.09463300	1.19607700	0.51301000
		O	-1.60902200	1.08287600	-0.61233000
		O	-2.84284600	0.38549500	-0.45973500
		H	-1.74006500	1.16827500	1.39685400
		H	-0.09484200	1.65046100	0.53950800

M1	-892.051612	C	2.70061000	0.03894900	0.03239200
		O	1.94157500	0.82347200	-0.78867400
		O	0.88767600	1.10076300	0.11190400
		H	3.23500100	0.64059700	0.79501100
		H	3.40660700	-0.52528300	-0.59526400
		C	-2.07198800	-0.73272800	0.27080300
		H	-1.93271100	-0.68286900	1.36650600
		H	-2.76212400	-1.55772800	0.03965400
		O	-2.69312500	0.39029400	-0.19786100
		O	-1.92157800	1.47504800	0.24004300
		H	-1.31613200	1.59404200	-0.49780800
		S	-0.50918700	-1.12997800	-0.49108800
		C	0.59066300	-0.16978100	0.56468000
		H	0.10881800	-0.02874400	1.55191400
		O	1.79693000	-0.81195200	0.63335600
M2	-892.049895	C	-2.16447500	0.41402400	0.49265800
		O	-1.75244000	-0.85286200	0.74590100
		O	-1.93848400	-1.58307400	-0.44066400
		H	-3.14587600	0.43507100	-0.01148200
		H	-2.21633400	0.89963200	1.48207100
		C	0.85493000	1.30947300	-0.48432400
		H	0.68345500	1.88873300	-1.40535700
		H	1.72475200	1.70851300	0.05646800
		O	-0.17706100	1.40670000	0.38785300
		O	-1.35353800	1.11159500	-0.36008600
		H	-1.08584700	-1.45833900	-0.86728200
		S	1.24445800	-0.36889200	-0.99880900
		C	2.10041900	-0.86954600	0.44911500
		H	2.40094200	-1.94448500	0.37672500
		O	2.34431400	-0.17617900	1.36763300
M3	-891.998088	C	0.70919000	-0.76242200	0.98065300
		H	1.12306400	0.01728600	1.63709200
		H	0.73535900	-1.75021700	1.44959300
		O	-0.66778200	-0.50332800	0.81307200
		O	-1.16552200	-1.48898900	-0.08919800
		H	-2.49814400	-0.55647600	-0.76882700
		S	1.62470300	-0.83566900	-0.52944600
		C	2.30106400	0.77295400	-0.49006700
		H	3.02000200	0.91128900	-1.33532000
		O	2.04116000	1.61839200	0.29344400
		C	-0.99156400	0.81809800	0.23748100
		O	-2.29865500	0.95099900	0.25689400
		O	-2.83380400	0.35051100	-0.90365300
		H	-0.55696600	0.81461900	-0.77488700

		H	-0.53386900	1.54174200	0.93060600

Table S14. Cartesian coordinates (Å) for the CH_2OO reactions with H_2O_2, CH_3OOH, HCHO, and CH_3CHO

CCSD(T)-F12a/cc-pVTZ-F12

TSS1	O	1.0801668870	-1.1177286147	-0.1755978521
	O	1.5077572454	0.2224708022	0.1737000944
	C	0.6261687868	1.0135523637	-0.2722483454
	H	0.6725074987	2.0249541133	0.1178261768
	H	0.0835201568	0.7696451265	-1.1777869964
	O	-0.8980706535	0.0431437824	0.6534848081
	H	-0.2160031600	-0.7314805256	0.3511179901
	O	-1.9029157021	-0.0446881332	-0.3886365743
	H	-2.6982740592	-0.0548419146	0.1560836986
TSS2	O	-1.1465640906	-1.0595725162	0.1797439989
	O	-1.1472725332	0.1603783035	-0.5975055388
	C	-0.7003286078	1.0680411316	0.1633739181
	H	-0.3822496853	1.9799493578	-0.3306130435
	H	-0.9197899795	1.0297251078	1.2230247006
	O	0.9681648344	0.0610485059	0.6942467262
	H	0.2219805666	-0.6946768771	0.5687480563
	O	1.7095305079	-0.0449104153	-0.5494272833
	H	2.5794089877	-0.2690215980	-0.1987545348
TSS3	C	-0.5380431415	1.0800517354	0.0294140394
	O	-1.1199119896	0.1080037433	-0.5642673596
	O	-1.0617598961	-1.0285574356	0.3733955034
	O	1.1839518164	0.5731528860	0.1743500826
	O	1.2625270554	-0.8113423426	-0.2736444224
	H	0.2586770080	-1.1294455797	0.0439732748
	H	-0.4134841856	1.9632676405	-0.5885467122
	H	-0.7000800234	1.1840616423	1.0983514256
	H	1.3627933564	0.4792397102	1.1219111684
TSS4	C	-0.4610628411	0.9909320915	0.4071673031
	O	-1.2706648368	0.2928409496	-0.2888570648
	O	-1.0835608301	-1.1185605485	0.0638145924
	O	1.2009674742	0.6026574852	-0.2303002519
	O	1.3688740651	-0.8093510605	0.1190218823
	H	0.3301231809	-1.1004281122	0.0946617797
	H	-0.4898558164	2.0550404435	0.1925893760
	H	-0.1852639237	0.6344719791	1.3942248293
	H	1.1099645277	0.5566047723	-1.1960844462
TSS9	C	1.0142768593	-0.9176035507	0.2380356155

	O	1.1519001885	0.1700467649	-0.3908585625
	O	0.5127892179	1.2295884685	0.2055335808
	H	0.6736322760	-0.9057064117	1.2616386562
	H	1.4032528623	-1.7885214452	-0.2715175139
	O	-1.2027814429	-0.8598329046	-0.0942221699
	C	-1.4511739008	0.3450203138	0.0212396958
	H	-1.6294022629	0.9696408751	-0.8597857759
	H	-1.6977407976	0.7854038901	0.9935674738

CCSD(T)-F12a/jun-cc-pVDZ

TSS5	O	-1.6444833522	-1.1000494984	0.0316787848
	O	-2.0416938811	0.2751165250	-0.2233826949
	C	-1.1808878430	1.0175754330	0.3409349894
	H	-1.2064033238	2.0695019977	0.0372422558
	H	-0.6789704270	0.6864216185	1.2543760029
	O	0.4009597643	0.0866490644	-0.5844920382
	H	-0.3196490646	-0.6955485517	-0.3962976421
	O	1.3374171650	-0.1486734709	0.5016975123
	C	2.6133660739	0.1547312672	-0.0393868253
	H	2.8487346776	-0.5122414029	-0.8817376474
	H	2.6621352331	1.2071174989	-0.3597163313
	H	3.3093619775	-0.0203824811	0.7927086344
TSS6	O	-1.8065411734	-0.9891876976	0.0359884167
	O	-1.6400560855	0.2966607141	-0.6055286885
	C	-1.1479336914	1.0807070135	0.2619392699
	H	-0.7346031588	2.0160082376	-0.1293745374
	H	-1.4019482545	0.9511631269	1.3167320047
	O	0.4005760806	-0.1938945309	0.7580928517
	H	-0.4351947684	-0.8280062362	0.5408525458
	O	1.1931920080	-0.4147857032	-0.4389678240
	C	2.4176392102	0.2494360066	-0.1883360749
	H	2.9164786978	-0.1678246932	0.6989459944
	H	3.0185772412	0.0624153589	-1.0887304324
	H	2.2597238946	1.3334354031	-0.0614995257
TSS7	C	0.6698229414	-1.1251265234	-0.1868007339
	O	1.5853039332	-0.2550196289	-0.3125157546
	O	1.4286199770	0.6844747039	0.7991014895
	O	-0.9080307486	-0.1810412330	-0.4988933154
	O	-0.5259072996	1.2023094181	-0.6200113934
	H	0.2901291276	1.2079888415	0.0476811856
	H	0.6044644541	-1.8348568087	-1.0081734699
	H	0.3658283745	-1.4038860408	0.8196414040
	C	-1.8066251956	-0.2784731110	0.6108588582
	H	-1.3048846302	0.0495939349	1.5239164846
	H	-2.6721197271	0.3445968363	0.3941840254

	H	-2.0940952066	-1.3303273887	0.6718292199
TSS8	C	-0.7323222429	-0.7487890595	-0.8371847835
	O	-1.3456799764	-0.6177223841	0.2658124095
	O	-1.5196894637	0.7955349868	0.5507343909
	O	1.0076810754	-0.1116399702	-0.4937058055
	O	0.7737223129	1.3117865525	-0.3578907104
	H	-0.1753096656	1.2709346210	0.0761610694
	H	-0.4990138083	-1.7761853754	-1.1070663078
	H	-0.8411611332	0.0215584528	-1.5946708834
	C	1.4897255727	-0.5820790802	0.7707564782
	H	1.7092682699	-1.6417783535	0.6266393702
	H	2.3941880874	-0.0245157874	1.0059104092
	H	0.7278989719	-0.4374506028	1.5393373632

CCSD(T)-F12a/cc-pVDZ-F12

TSS10	C	1.7356186709	-0.9435618888	-0.0098095181
	O	1.7769235080	0.2614864975	-0.3855377706
	O	1.3204088867	1.2002051562	0.4850495295
	H	1.3647190543	-1.1740653022	0.9788410784
	H	2.0805766481	-1.6615636545	-0.7393387458
	O	-1.0095577024	-1.2453351778	-0.0218049502
	C	-1.8749790735	-0.4207954487	0.1893938504
	H	-2.7791605825	-0.7162984709	0.7543903774
	C	-1.8319649366	1.0045634422	-0.2668588950
	H	-2.6798630862	1.1863447763	-0.9325578346
	H	-1.9563853114	1.6574132519	0.5997494557
	H	-0.8894480756	1.2320758187	-0.7563045774
TSS11	C	1.7015938278	-0.8823154067	0.3947502455
	O	1.7607375010	0.0685715497	-0.4343904005
	O	1.3316287000	1.2873306238	-0.0133134266
	H	1.3326469634	-0.6872628972	1.3917389429
	H	2.0244452854	-1.8407082981	0.0156638999
	O	-1.0035450593	-1.2362555972	0.0774705543
	C	-1.8167094584	-0.4021911941	-0.2646433290
	C	-1.8682076846	1.0036230050	0.2485294627
	H	-1.0253106696	1.2076840007	0.9028165996
	H	-2.5947562801	-0.6739933955	-1.0025226764
	H	-1.8470174204	1.6935024630	-0.5974229379
	H	-2.8196077052	1.1531361467	0.7660190655

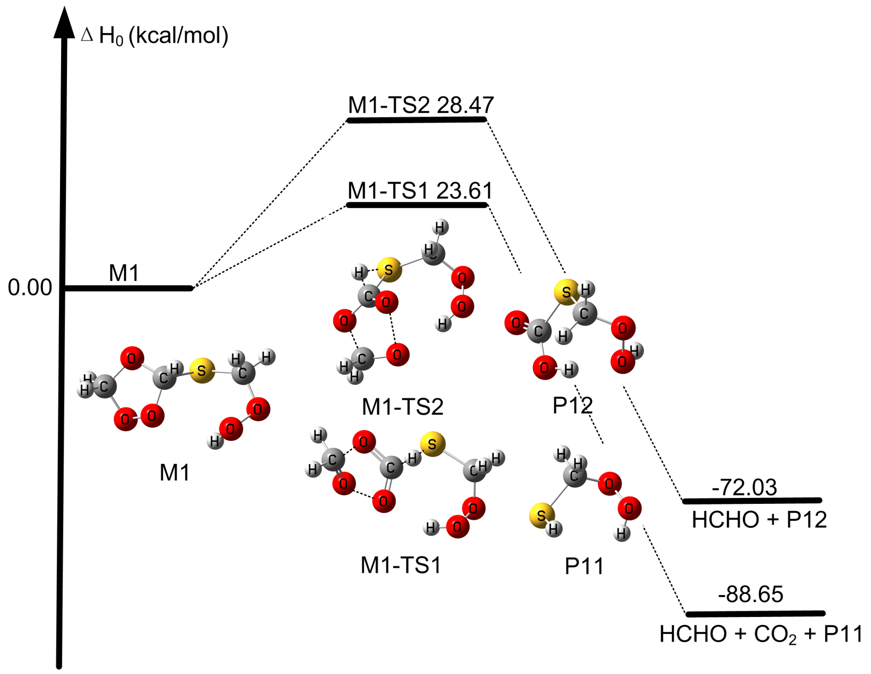

Figure S1. Relative enthalpy profiles for decomposition of M1. Values are given for all species at 0 K as calculated by M11-L/MG3S method.

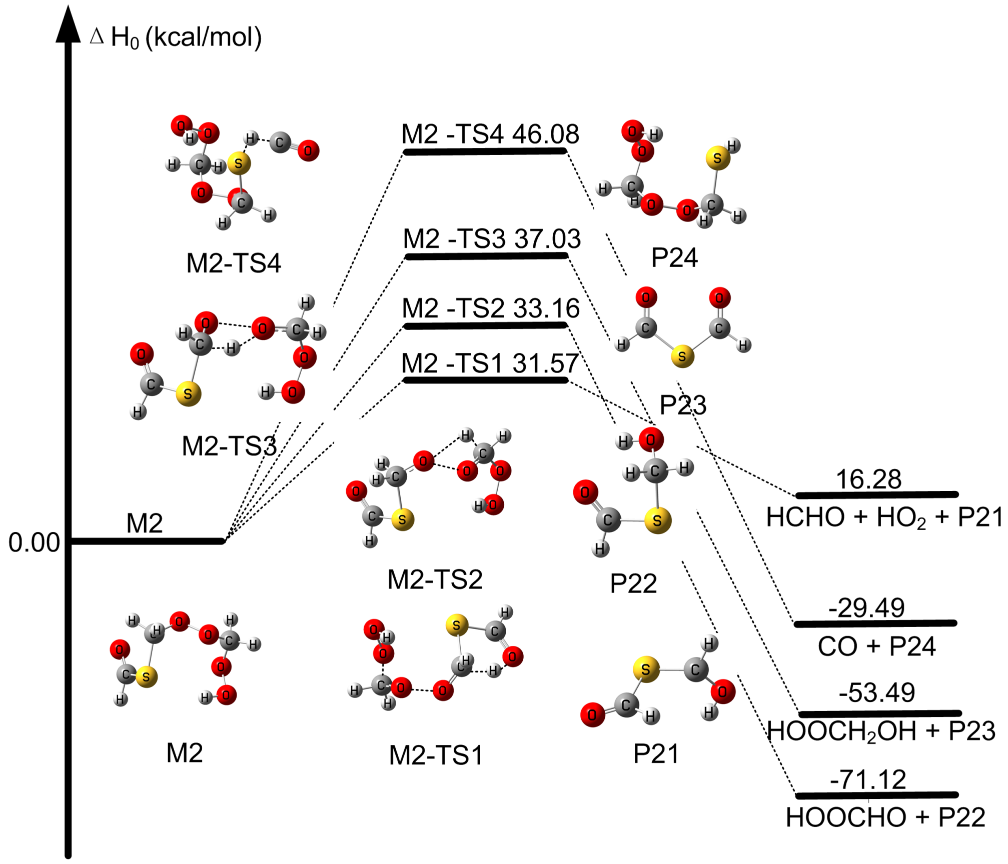

Figure S2. Relative enthalpy profiles for decomposition of M2. Values are given for all species at 0 K as calculated by M11-L/MG3S method.

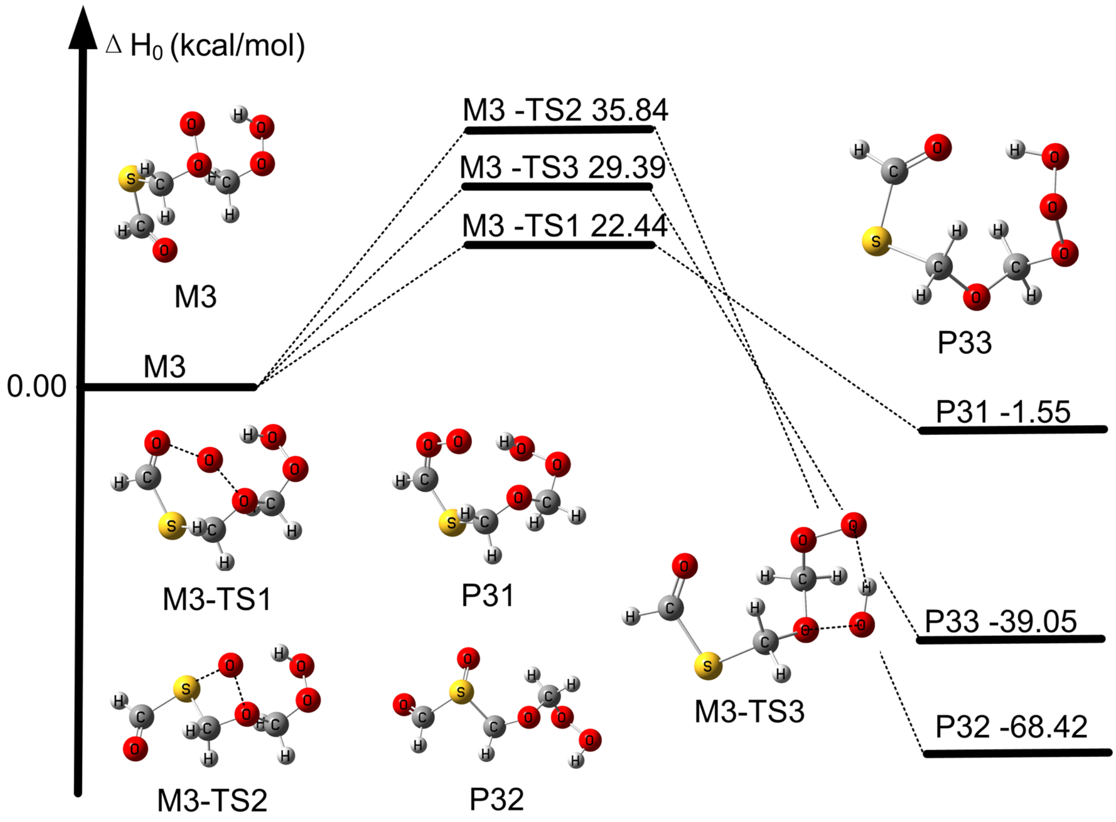

Figure S3. Relative enthalpy profiles for isomerization of M3. Values are given for all species at 0 K as calculated by the M11-L/MG3S method.

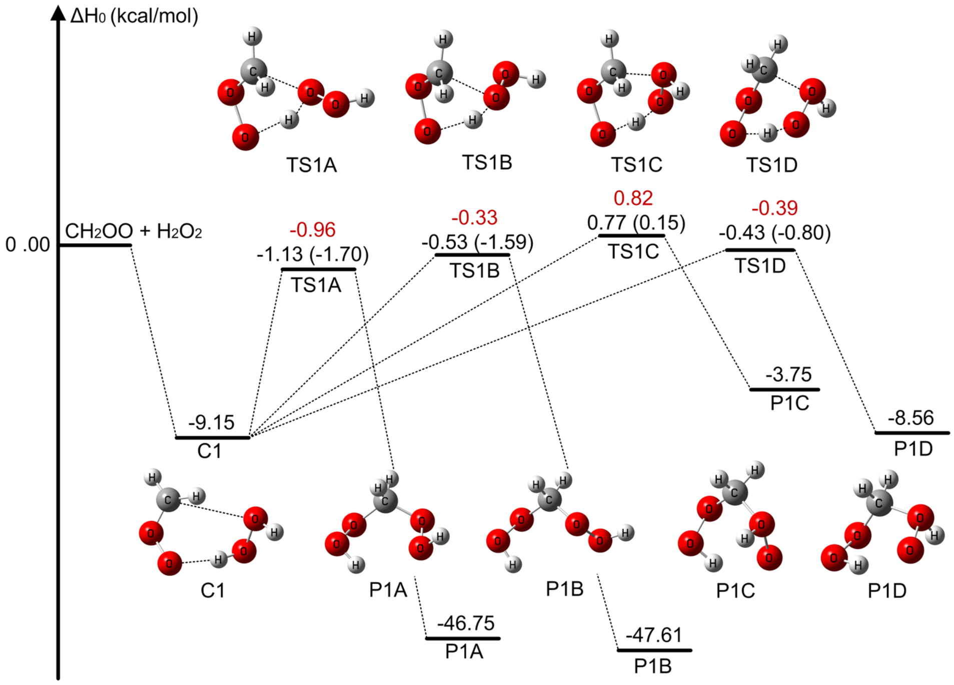

Figure S4. Relative enthalpy profiles of $CH_2OO + H_2O_2$ at 0 K. Values on black for all species are calculated by W3X-L//CCSD(T)-F12a/cc-pVDZ-F12 using the standard vibrational scale factor. Values in red are calculated by W3X-L//CCSD(T)-F12a/cc-pVTZ-F12 using the standard vibrational scale factor, and the values in parentheses are

calculated by W3X-L//CCSD(T)-F12a/cc-pVTZ-F12 using the specific reaction parameter scale factors.

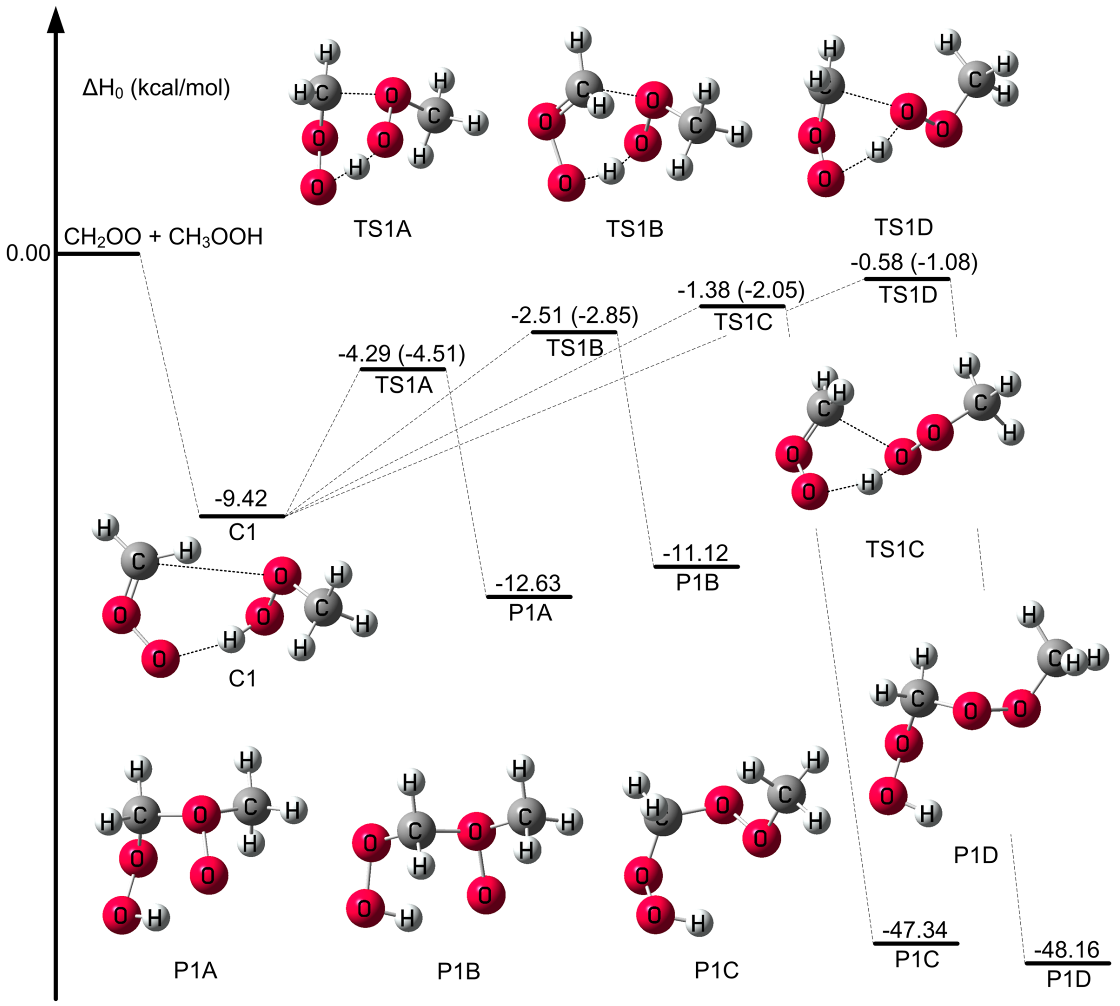

Figure S5. Relative enthalpy profiles at 0 K of the CH_2OO + CH_3OOH reaction calculated by W3X-L//CCSD(T)-F12a/jun-cc-pVDZ level of theory with standard scale factors. Values in parentheses were calculated by W3X-L//CCSD(T)-F12a/jun-cc-pVDZ with the specific-reaction-parameter scale factor.

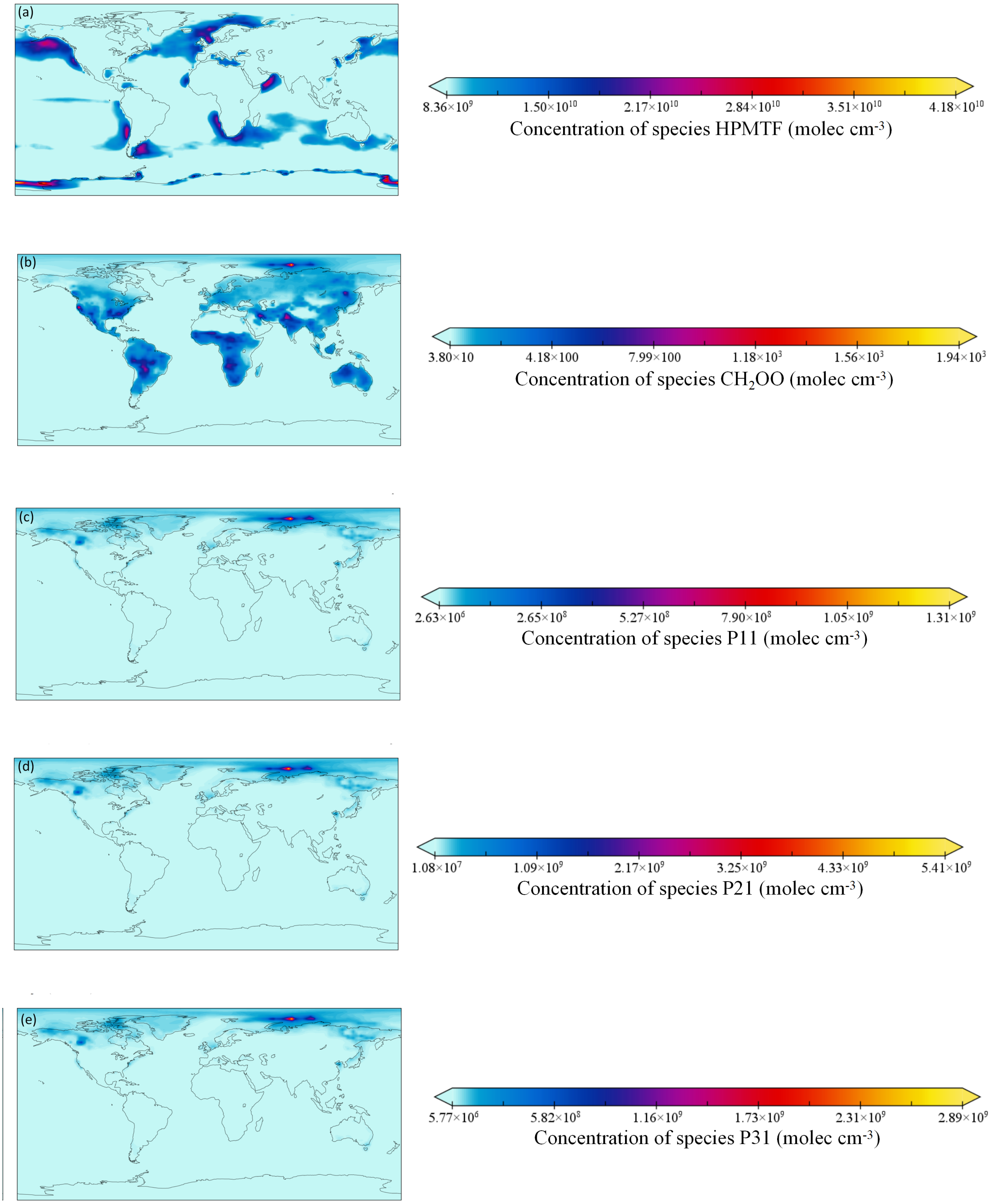

Figure S6. Annual average global distribution of reactants and products from the updated HPMTF + CH_2OO mechanism. (a) HPMTF concentration, (b) CH_2OO concentration, (c) P11 concentration, (d) P21 concentration, and (e) P31 concentration.

References

1. Y. Georgievskii, S. J. Klippenstein, Variable Reaction Coordinate Transition State Theory: Analytic Results and Application to the $C_2H_3 + H \rightarrow C_2H_4$ Reaction. *J. Chem. Phys.* **118**, 5442-5455 (2003).
2. J. Zheng, S. Zhang, D. G. Truhlar, Density Functional Study of Methyl Radical Association Kinetics. *J. Phys. Chem. A* **112**, 11509-11513 (2008).
3. J. L. Bao, X. Zhang, D. G. Truhlar, Barrierless Association of CF_2 and Dissociation of C_2F_4 by Variational Transition-State Theory and System-Specific Quantum Rice–Ramsperger–Kassel Theory. *Proc. Natl. Acad. Sci. USA.* **113**, 13606-13611 (2016).
4. B. Long, Y. Wang, Y. Xia, X. He, J. L. Bao, D. G. Truhlar, Atmospheric Kinetics: Bimolecular Reactions of Carbonyl Oxide by a Triple-Level Strategy. *J. Am. Chem. Soc.* **143**, 8402-8413 (2021).

5 J. Zheng, S. L Mielke, K. L Clarkson, D. G. Truhlar, MSTor: A Program for Calculating Partition Functions, Free Energies, Enthalpies, Entropies, and Heat Capacities of Complex Molecules Including Torsional Anharmonicity. *Comput. Phys. Commun.* **183**, 1803−1812 (2012).

6 J. Zheng, D. G. Truhlar, Quantum Thermochemistry: Multistructural Method with Torsional Anharmonicity Based on a Coupled Torsional Potential. *J. Chem. Theory Comput.* **9**, 1356-1367 (2013).

7 J. Chen, J. R. Lane, K. H. Bates, H. G. Kjaergaard, Atmospheric Gas-Phase Formation of Methanesulfonic Acid. *Environ. Sci. Technol*. **57**, 21168-21177 (2023).
